# Peripheral *N*-methyl-d-aspartate receptor activation contributes to monosodium glutamate-induced headache but not nausea behaviours in rats

**DOI:** 10.1038/s41598-022-18290-w

**Published:** 2022-08-16

**Authors:** Tarique Benbow, Felisha Teja, Afrooz Sheikhi, Fernando G. Exposto, Peter Svensson, Brian E. Cairns

**Affiliations:** 1 Faculty of Pharmaceutical Sciences, University of British Columbia, 2405 Wesbrook Mall, Vancouver, V6T 1Z3 Canada; 2 Section for Orofacial Pain and Jaw Function, Department of Dentistry and Oral Health, Aarhus University, Vennelyst Boulevard 9, 8000 Aarhus C, Denmark

**Keywords:** Neuroscience, Neurology

## Abstract

Monosodium glutamate induces behaviors thought to reflect headache and nausea in rats. We explored the effects of the *N*-methyl-d-aspartate receptor antagonist (2R)-amino-5-phosphonovaleric acid, the inotropic glutamate receptor antagonist kynurenic acid, and the CGRP receptor antagonist olcegepant, on monosodium glutamate-induced increases in nocifensive, headache-like and nausea behaviours. Effects of these antagonists on motor function were examined with a rotarod. The effect of the dopamine receptor antagonist metoclopramide and the serotonin 3 receptor antagonist ondansetron on nausea behaviour was also assessed. (2R)-amino-5-phosphonovaleric acid, and to a lesser extent, kynurenic acid and olcegepant, reduced nocifensive and headache-like behaviours evoked by monosodium glutamate. No alteration in motor function by (2R)-amino-5-phosphonovaleric acid, kynurenic acid or olcegepant was observed. No sex-related differences in the effectiveness of these agents were identified. Nausea behaviour was significantly more pronounced in male than in female rats. Olcegepant, ondansetron and metoclopramide ameliorated this nausea behaviour in male rats. Ondansetron and metoclopramide also reduced headache-like behaviour in male rats. These findings suggest that peripheral *N*-methyl-d-aspartate receptor activation underlies monosodium glutamate-induced headache-like behaviour but does not mediate the nausea behaviour in rats.

## Introduction

Peripheral glutamate signalling has been proposed to play a role in the initiation of migraine headaches^1^. Consumption of monosodium glutamate (MSG: 150 mg/kg) results in reports of headache, craniofacial sensitivity and nausea in healthy humans^2^. Blood levels of glutamate are also elevated during and after a migraine headache^3,4^. Furthermore, genome wide association studies have shown significant polymorphisms in glutamate receptor and transporter genes that are associated with migraine^5^. Functional studies in rats have delineated that elevated blood levels of glutamate dilate dural blood vessels and increase the response of trigeminovascular neurons to mechanical stimulation of the dura^6^. Altogether, these findings suggest that a disruption in peripheral glutamate homeostasis may contribute to migraine headaches and some of their associated symptoms^1^.

We have developed an in vivo rat model, which uses systemic administration of MSG to induce headache-like and nausea behaviours^7^. This model produces dose- and time-dependent increases in nocifensive and headache-like behaviours in a sexually dimorphic fashion. Specifically, intraperitoneal injections (i.p.) of 1000 mg/kg MSG significantly increased nocifensive and headache-like behaviours compared to control (saline), which are attenuated by administration of the migraine abortive therapy sumatriptan^7^. In addition, this dose of MSG produces behavioural and physiological signs of nausea in rats. Headache-like and nausea behaviours occur in association with an increase in plasma glutamate and calcitonin-gene related peptide (CGRP) concentrations, supporting the notion that peripheral glutamate receptor activation may directly or indirectly (through CGRP-mediated mechanisms) contribute to headache pathogenesis^1,7^. Nevertheless, the specific excitatory amino acid receptors through which peripheral glutamate contributes to headache generation in this model have not been identified.

We hypothesized that peripheral glutamate receptor activation contributes to nocifensive and headache-like behaviour in this rat model of migraine. In the present study, we utilized (2R)-amino-5-phosphonovaleric acid (APV) a selective competitive NMDA receptor antagonist and kynurenic acid (KYNA) a non-competitive NMDA and competitive AMPA/Kainate receptor antagonist in the MSG-induced headache model to determine which type of peripheral ionotropic glutamate receptors may be responsible for the behaviours described above. These glutamate receptor antagonists were specifically chosen because they have very poor central nervous system penetration^8–10^. We also investigated whether the downstream effects of peripheral glutamate receptor activation on headache-like and nocifensive behaviours involve CGRP receptor activation by utilizing the CGRP receptor antagonist olcegepant. Finally, we validated MSG-induced nausea behaviour through the use of the antiemetic drugs metoclopramide (dopamine receptor antagonist; MTC) and OND (serotonin 5-HT_3_ antagonist).

## Results

### The effects of glutamate- and CGRP-receptor antagonists on nocifensive- and spontaneous headache-like behaviours evoked by MSG

**Figure 1:**
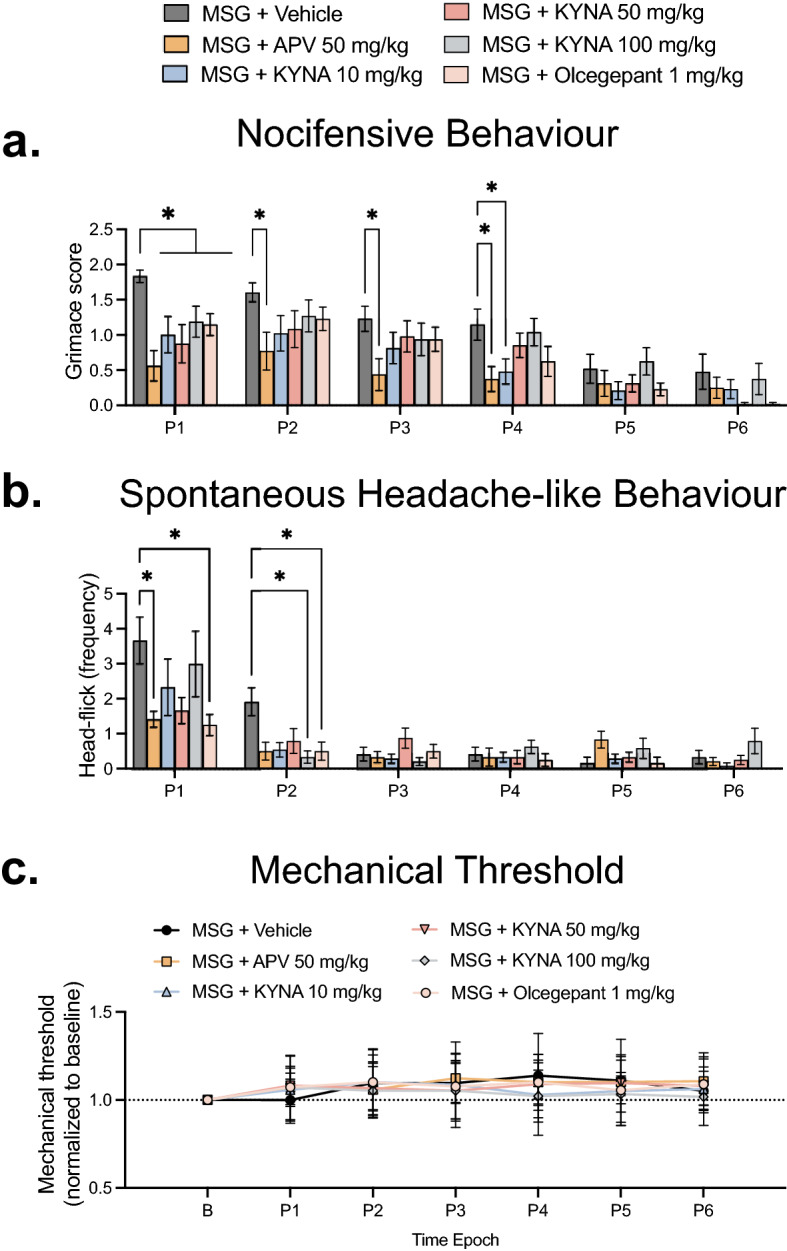
APV, KYNA and Olcegepant attenuate MSG-induced nocifensive and headache-like behaviours. Comparison of the effect of MSG (1000 mg/kg; i.p.) alone compared with APV (50 mg/kg; i.p.), KYNA (10, 50, 100 mg/kg; i.p.) and olcegepant (1 mg/kg; i.p.) in combination with MSG (1000 mg/kg; i.p.) on (**a**) Grimace scores, (**b**) Head-flick frequency and (**c**) Temporalis muscle MT. Note: APV significantly attenuated MSG-induced nocifensive behaviour over the first 40 min, while also significantly decreasing MSG-induced head-flick frequency during the first 10 min compared to MSG alone. KYNA at all doses decreased MSG-induced nocifensive behaviour during the first 10 min, and the highest dose (100 mg/kg) also reduced head-flick frequency from 10 to 20 min compared to MSG alone. Olcegepant reduced MSG-induced nocifensive behaviour for the first 10 min, and head-flick frequency for the first 20 min compared to MSG alone. There was no significant effect of any of the treatments on MT. Data are mean ± SEM (n = 12) *p < 0.05, repeated measures two-way ANOVA and Dunnett’s test. Time Epoch = P1 (0–10 min), P2 (10–20 min), P3 (20–30 min), P4 (30–40 min), P5 (40–50 min) and P6 (50–60 min) post injection.

We investigated whether NMDA, non-NMDA or CGRP receptors contribute to nocifensive, and headache-like behaviours elicited by MSG. There was a significant effect of time (F_(3.486, 230.1)_ = 43.53; *p* < 0.0001), treatment (F_(5, 66)_ = 28.53; *p* = 0.0204) and time/treatment interaction (F_(25, 330)_ = 1.681; *p* = 0.0234) on grimace scores (Fig. 1a). It was found that APV (50 mg/kg i.p.) significantly reduced grimace score compared to vehicle at P1–P4 (*p* < 0.05) (Fig. 1a). KYNA (10 mg/kg, 50 mg/kg, and 100 mg/kg; i.p.) significantly reduced the grimace score when compared to vehicle at P1 (*p* < 0.05). KYNA (10 mg/kg; i.p.) also significantly reduced grimace score compared to vehicle at P4 (*p* < 0.0313). Olcegepant (1 mg/kg; i.p.) significantly reduced the grimace score when compared to vehicle at P1 (*p* = 0.0199) (Fig. 1a). There were no significant sex-related differences observed on grimace scores across the different treatment groups (*p* > 0.05).

There was a significant effect of time (F_(5, 66)_ = 25.24; *p* < 0.0001), treatment (F_(3.993, 263.5)_ = 4.031; *p* = 0.0035) and time/treatment interaction (F_(25, 330)_ = 2.310; *p* = 0.0005) on head-flick frequency. APV (50 mg/kg; i.p.) significantly attenuated head-flick frequency evoked by MSG at P1 (*p* = 0.0212) (Fig. 1b). KYNA (100 mg/kg; i.p.) significantly reduced head-flick frequency elicited by MSG (1000 mg/kg; i.p.) at P2 (*p* = 0.0248) (Fig. 1b). Olcegepant (1 mg/kg; i.p.) significantly attenuated the head-flick frequency evoked by MSG (1000 mg/kg; i.p.) at P1 (*p* = 0.0295) and P2 (*p* = 0.0359) (Fig. 1b). There were no significant sex-related differences in the effect of any of these treatments on MSG-evoked increased head-flick frequency. There was also no significant effect of any treatment on temporalis mechanical threshold (MT) in these experiments (Fig. 1c).

### Effect of NMDA- and CGRP- receptor antagonists on MSG-induced suppression of facial grooming and normal exploratory behaviour

**Figure 2:**
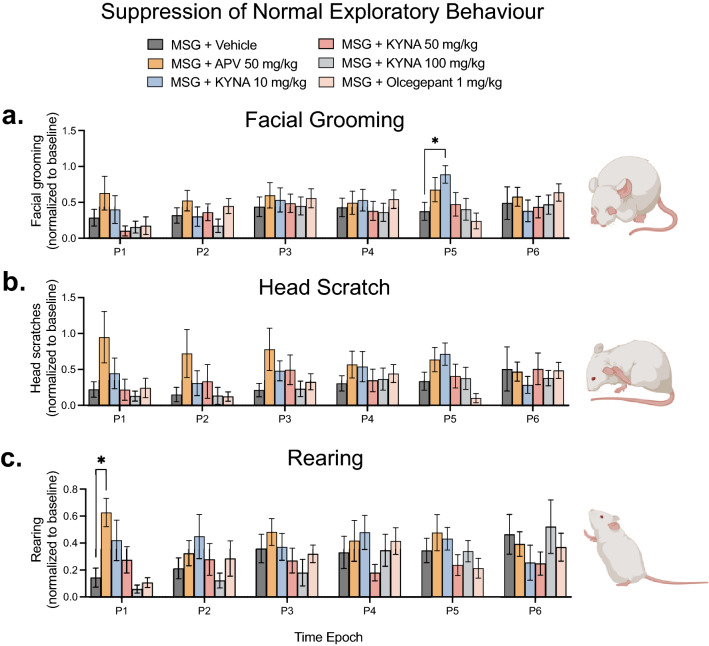
APV, KYNA and Olcegepant had no effect on MSG-induced suppression of normal exploratory and grooming behaviours. Comparison of the effect of MSG (1000 mg/kg; i.p.) alone with APV (50 mg/kg; i.p.), KYNA (10, 50, 100 mg/kg; i.p.) and olcegepant (1 mg/kg; i.p.) in combination with MSG (1000 mg/kg; i.p.) on (**a**) Facial grooming (**b**) Head scratch and (**c**) Rearing behaviours. Treatments had little effect on MSG-induced suppression of normal exploratory and grooming behaviours. The suppression of facial grooming was only significantly attenuated by KYNA (10 mg/kg) at P5. The suppression of rearing behaviour was significantly attenuated by APV at P1. Data are mean ± SEM (n = 12). Repeated measures two-way ANOVA and Dunnett’s test. Time Epoch = P1 (0–10 min), P2 (10–20 min), P3 (20–30 min), P4 (30–40 min), P5 (40–50 min) and P6 (50–60 min) post injection. (Created with personal license of BioRender).

We have previously found that MSG (1000 mg/kg, i.p.) significantly suppresses normal exploratory and grooming (facial grooming and head scratch) behaviours compared to saline control^7^. There was a significant effect of time (F_(4.510, 297.7)_ = 3.088; *p* = 0.0126), but no significant effect of treatment (F_(5, 66)_ = 1.365; *p* = 0.2487) or time/treatment interaction (F_(25, 330)_ = 0.9124; *p* = 0.5883) for facial grooming behaviour (Fig. 2a). KYNA (10 mg/kg; i.p.) significantly increased facial grooming behaviour compared to MSG alone at P5 (*p* = 0.0313; Fig. 2a). There was no significant effect of time (F_(3.521, 232.4)_ = 0.9913; *p* = 0.4064), treatment (F_(5, 66)_ = 1.702; *p* = 0.1464) or time/treatment interaction (F_(25, 330)_ = 1.091; *p* = 0.3509) for head scratch behaviour. Although there was a trend towards APV (50 mg/kg; i.p.) increasing relative head scratch behaviours from P1—P5, this was not statistically significant (Fig. 2b). There was a significant time/treatment interaction (F_(25, 330)_ = 1.847; *p* = 0.0091) but no significant effect of time (F_(3.493, 230.5)_ = 1.612; *p* = 0.1797) and treatment (F_(5, 66)_ = 1.157; *p* = 0.3396) on rearing behaviour (Fig. 2c). APV (50 mg/kg; i.p.) significantly attenuated the suppression of rearing behaviour by MSG at P1 (*p* = 0.005) (Fig. 2c). Neither KYNA nor olcegepant had an effect on normal rearing behaviour. There were no significant sex-related differences observed for any of the treatments.

### Olcegepant attenuates MSG-induced nausea behaviour

**Figure 3:**
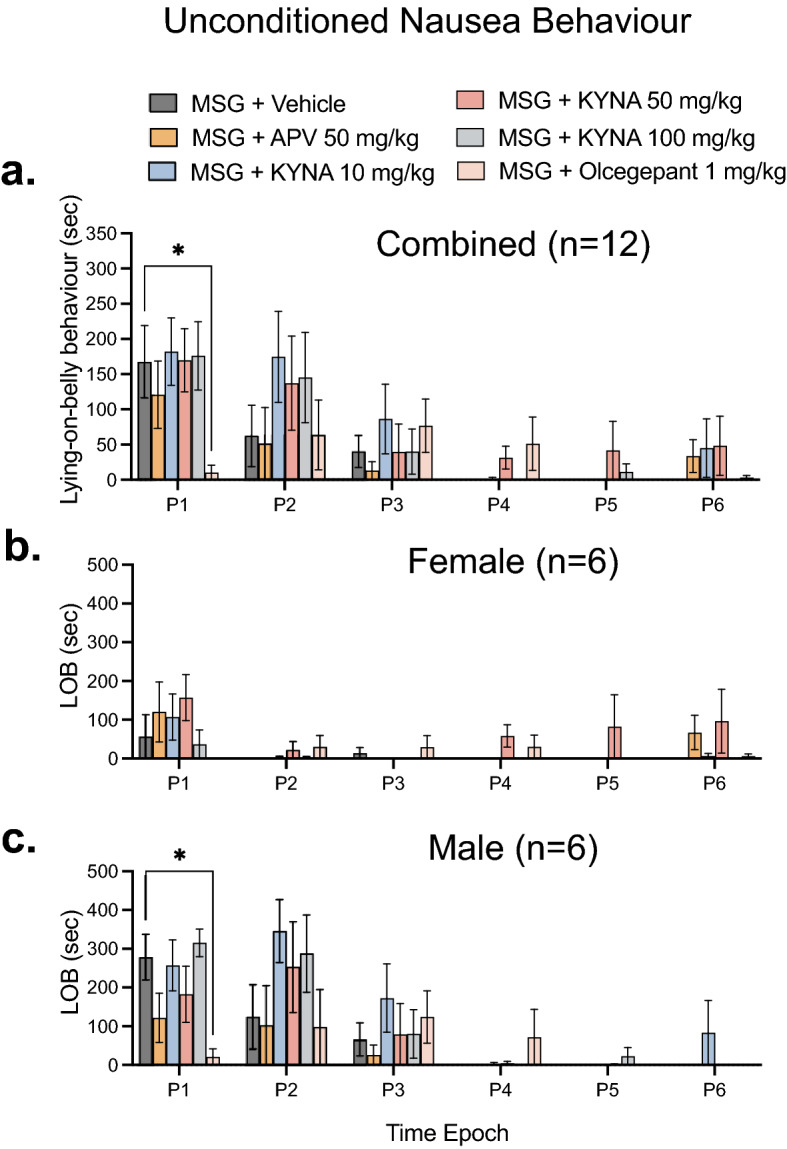
Olcegepant attenuated MSG-induce nausea behaviours in male rats. Comparison of the effect of MSG (1000 mg/kg; i.p.) alone with APV (50 mg/kg; i.p.), KYNA (10, 50, 100 mg/kg; i.p.) and olcegepant (1 mg/kg; i.p.) in combination with MSG (1000 mg/kg; i.p.) on LOB behaviour duration in (**a**) Males and Females (n = 12) (**b**) Females only (n = 6) (**c**) Males only (n = 6). Only olcegepant administration was shown to significantly reduce this behaviour in male rats. Data are mean ± SEM (n = 6). *p < 0.05. Time Epoch = P1 (0–10 min), P2 (10–20 min), P3 (20–30 min), P4 (30–40 min), P5 (40–50 min) and P6 (50–60 min) post injection.

We also explored whether APV, KYNA or olcegepant could attenuate the increased Lying-on-belly (LOB) behaviour duration induced by MSG. A significant effect of treatment (F_(3.923, 258.9)_ = 3.184; *p* = 0.0147), time (F_(5, 66)_ = 4.756; *p* = 0.0009) and interaction (F_(25, 330)_ = 1.623; *p* = 0.0321) for MSG induced LOB behaviour was found. Only olcegepant (1 mg/kg; i.p.) significantly inhibited MSG induced LOB duration when compared to vehicle at P1 (*p* = 0.0301) (Fig. 3a). Consistent with our previous results, males were found to exhibit a substantially greater increase in LOB duration compared to females^7^. In females, there was no significant effect of MSG treatment on LOB behaviour (Fig. 3b). In males, there was a significant effect of treatment (F _(3.869, 116.1)_ = 4.456; *p* = 0.0025), time (F_(5, 30)_ = 5.773; *p* = 0.0008) and time /treatment interaction (F_(25, 150)_ = 1.923; *p* = 0.0089) for LOB behaviour (Fig. 3c). Post-hoc Dunnett’s analysis revealed that olcegepant (1 mg/kg; i.p.) significantly inhibited MSG induced LOB duration when compared to vehicle at P1 in male rats (*p* = 0.0192) (Fig. 3c). In contrast, there was a non-significant trend towards increased LOB behaviour in male rats treated with KYNA.

### Systemic administration of KYNA, APV and olcegepant does not alter motor co-ordination or balance

**Figure 4:**
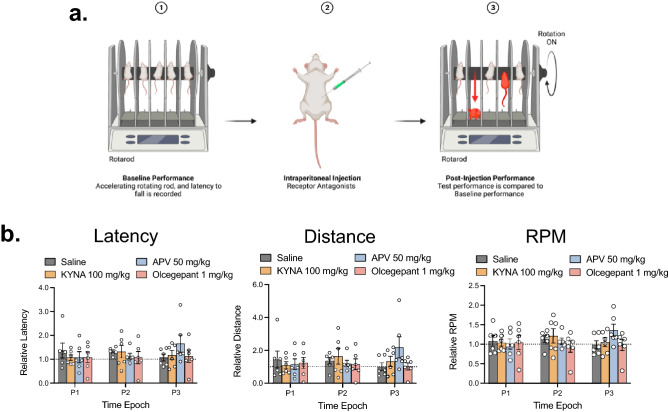
Systemic administration of APV, KYNA and Olcegepant did not induce motor function or balance deficits. Comparison of the effect of APV (50 mg/kg; i.p.), KYNA (10, 50, 100 mg/kg; i.p.), olcegepant (1 mg/kg; i.p.) and vehicle on motor coordination and balance in 3 male and 3 female rats. (**a**) Schematic diagram illustrating the accelerated rotarod protocol used to assess motor coordination and balance. (**b**) The bar charts show the lack of effect of systemic administration of APV, KYNA and olcegepant on the mean relative latency, distance travelled and speed (RPM). Data are mean ± SEM (n = 6). Symbols represent individual data points. *P < 0.05. Time Epoch = P1 (0–10 min), P2 (10–20 min) and P3 (20–30 min). (Created with personal license of BioRender).

To investigate whether systemically administered ionotropic glutamate receptor antagonists (KYNA and APV) or the CGRP receptor antagonist (olcegepant) produced any central nervous system effects, we assessed motor function and balance post administration by using an accelerated rotarod protocol (ACP; Fig. 4a). There was no significant effect of treatment, time, or time/treatment interaction for rotarod latency, distance, and speed (RPM) post treatment (*p* > 0.05). It was found that neither KYNA (100 mg/kg; i.p.), APV (50 mg/kg; i.p.) nor olcegepant (1 mg/kg; i.p.) reduced latency, distance travelled and speed when compared to baseline in the ACP (Fig. 4b). Thus, there was no evidence of loss of motor coordination or balance dysfunction following treatment of KYNA, APV and olcegepant.

### Ondansetron and metoclopramide attenuate MSG-induced lying-on-belly behaviours

**Figure 5:**
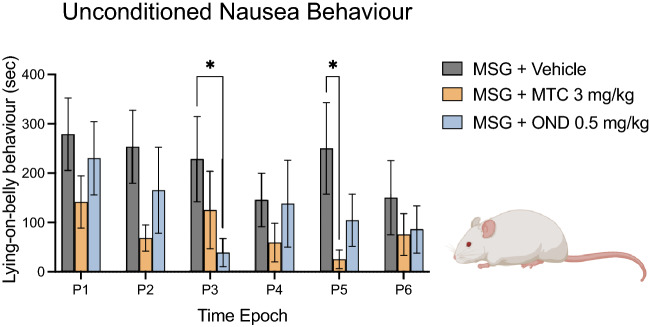
OND and MTC ameliorated MSG—induced nausea behaviours in male rats. The effects of MTC (3 mg/kg; i.p.), OND (0.5 mg/kg; i.p.) and vehicle in combination with MSG (1000 mg/kg; i.p.) on MSG induced LOB behaviour duration in 6 male rats. Both MTC and OND lowered LOB behaviour, but significant changes occurred at P3 and P5, for each agent, respectively. Data are mean ± SEM (n = 6). *P < 0.05. Time Epoch = P1 (0–10 min), P2 (10–20 min), P3 (20–30 min), P4 (30–40 min), P5 (40–50 min) and P6 (50–60 min) post injection.

To validate that MSG-induced LOB is reflective of nausea, we investigated the effects of co-administration of MSG (1000 mg/kg; i.p.) with MTC (3 mg/kg; i.p.) and OND (0.5 mg/kg; i.p.). These experiments were conducted in a separate group of male *Sprague Dawley* rats (n = 6), since we found that male rats demonstrated significantly longer duration of LOB behaviours compared to female rats (Fig. 3)^7^. There was a significant effect of treatment (F_(2, 60)_ = 8.462; *p* = 0.0006), but no significant effect of time (F_(5, 30)_ = 0.8777; *p* = 0.5079) or time/treatment interaction (F_(10, 60)_ = 0.6457; *p* = 0.7688) for LOB duration (Fig. 5). Although both treatments attenuated LOB behaviour compared to MSG alone for the duration of the experiment, it was found that OND (0.5 mg/kg; i.p.) only significantly attenuated LOB duration induced by MSG (1000 mg/kg; i.p.) when compared to vehicle at P3 (*p* = 0.045). Similarly, we found that MTC (3 mg/kg; i.p.) only significantly attenuated LOB duration induced by MSG (1000 mg/kg; i.p.) when compared to vehicle at P5 (*p* = 0.0153).

### Effects of OND and MTC on nocifensive-, headache-like and suppression of normal exploratory behaviours induced by MSG

**Figure 6:**
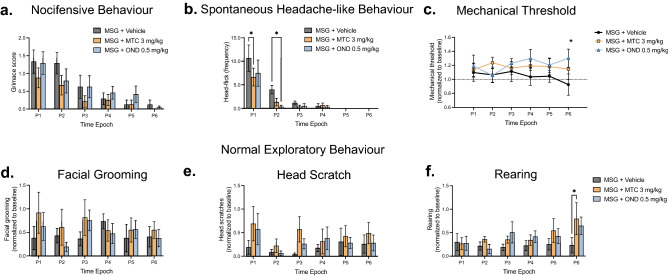
OND and MTC ameliorated MSG induced headache-like behaviours but not nocifensive or exploratory behaviours. The effects of MTC (3 mg/kg; i.p.), OND (0.5 mg/kg; i.p.) and vehicle in combination with MSG (1000 mg/kg; i.p.) on (**a**) Grimace scores (**b**) Head-flick frequency (**c**) Mechanical withdrawal threshold (OND vs Vehicle *P < 0.05) (**d**) Facial grooming (**e**) Head scratches and (**f**) Rearing in 6 male rats. Both MTC and OND reduced head flick frequency. MTC also had a late effect on MSG-induced suppression of rearing behaviour. However, neither MTC nor OND had a significant effect on nocifensive or grooming behaviours. Data are mean ± SEM (n = 6). *P < 0.05. Time Epoch = P1 (0–10 min), P2 (10–20 min), P3 (20–30 min), P4 (30–40 min), P5 (40–50 min) and P6 (50–60 min) post injection.

We also investigated the effects of MTC and OND on MSG-induced nocifensive- and headache-like behaviours. There was a significant effect of time (F_(5, 30)_ = 9.029; *p* < 0.0001) but not treatment (F_(2, 60)_ = 2.736; *p* = 0.0729) or time/treatment interaction (F_(10, 60)_ = 0.5284; *p* = 0.8634) for grimace scores. We found that compared to vehicle, neither MTC (3 mg/kg; i.p.) nor OND (0.5 mg/kg; i.p.) had a significant effect on grimace scores compared with MSG alone (Fig. 6a). There was a non-significant trend towards MTC lowering grimace scores when compared to vehicle from P1 to P3. There was a significant effect of time (F_(5, 75)_ = 29.09; *p* < 0.0001), but no significant effect of treatment (F_(2, 15)_ = 1.567; *p* = 0.2410) or time/treatment interaction (F_(10, 75)_ = 0.9698; *p* = 0.4766) for head-flick frequency (Fig. 6b). Post-hoc Dunnett’s analysis demonstrated that MTC and OND significantly reduced the frequency of head-flick behaviours when compared to vehicle at P1 (*p* = 0.0226) and P2 (*p* = 0.0389), respectively (Fig. 6b). There was a significant effect of treatment (F_(2, 60)_ = 3.432; *p* = 0.0388), but no significant effect of time (F_(5, 30)_ = 0.1189; *p* = 0.9872) or time/treatment interaction (F_(10, 60)_ = 0.4611; *p* = 0.9083) for temporalis MT (Fig. 6c). We identified a time-dependent increase in temporalis muscle region MT when MSG was co-administered with OND (0.5 mg/kg; i.p.) compared to vehicle at P6 (*p* = 0.0456) (Fig. 6c). We evaluated the effects of MTC (3 mg/kg; i.p) and OND (0.5 mg/kg; i.p.) in our experimental paradigm on MSG induced suppression of facial grooming, head scratches and normal exploratory behaviours. No significant effects on MSG-suppressed normal exploratory or grooming behaviours were identified (*p* > 0.05) (Fig. 6d and 6e). However, Dunnett’s post-hoc analysis indicated that MTC (3 mg/kg; i.p.) significantly attenuated MSG-suppressed rearing behaviour at P6 (p = 0.0326) (Fig. 6f).

## Discussion

In this study we examined whether peripheral glutamate receptor activation contributes to changes in nocifensive and headache-like behaviours in a rat model of headache with migraine-like symptoms. We found that MSG induces nocifensive- and headache-like behaviours principally through activation of peripheral NMDA receptors. This interpretation is supported by the significant inhibitory effect of APV on headache-like behaviors, and by the finding that the glutamate inotropic receptor antagonist KYNA was no more effective at reducing these behaviours. Additionally, MSG-induced nocifensive and headache-like behaviours were partly mediated through activation of CGRP receptors, as they were attenuated by olcegepant, a selective CGRP receptor antagonist. This finding bolsters the argument that systemic administration of MSG in rats is a valid model of migraine-like headache, as olcegepant has been shown to be an effective migraine abortive drug in humans and other preclinical models of migraine^11–14^. It also suggests that elevated CGRP levels contribute to the changes in behaviour observed after MSG administration^7^. The suppression of normal exploratory behaviour (rearing) is likely secondary to ongoing glutamate-induced pain, as it was also attenuated by the selective NMDA receptor antagonist APV.

While KYNA at higher doses was able to suppress MSG-induced nocifensive and headache-like behaviours, a dose response relationship for these effects was not identified in the present study. KYNA is an endogenous non-competitive antagonist of the NMDA receptor that only poorly penetrates the blood brain barrier, which suggests that its effects are mediated peripherally. In chronic migraineurs, serum levels of KYNA are reduced, which has been suggested to contribute to overactivity of NMDA receptors in migraine^7,15,16^. Based on these findings, it has been proposed that inhibition of NMDA receptor activation may be effective for migraine prophylaxis, although inhibition of central nervous system NMDA receptors by increasing KYNA in the brain has been the focus. Indeed, both preclinical and phase 1 studies have explored the utility of the KYNA precursor kynurenine in this regard^17,18^. Our results indicate that doses of 50 mg/kg or higher of KYNA were effective in the MSG headache model without causing centrally mediated effects. This suggests KYNA may have potential as a peripherally restricted migraine prophylactic therapy.

As previously reported, only male rats exhibited significant LOB behaviour, which is thought to reflect ongoing nausea^7^. Both MTC and OND attenuated MSG-induced nausea behaviour, which provides evidence that LOB is a valid indication of ongoing nausea in this model. While there was no effect of glutamate receptor antagonists on this behaviour, olcegepant did significantly attenuate LOB, suggesting that CGRP receptor activation partly underlies it. MTC, an antiemetic drug which is also used as a migraine abortive agent, was found to attenuate spontaneous head-flick behaviour, adding further evidence that this behaviour is as a valid representation of headache in rats. These findings support a conclusion that administration of 1000 mg/kg MSG to Sprague Dawley rats produces several migraine headache-like symptoms, only some of which can be attenuated by inhibition of peripheral glutamate receptors.

Previously we showed that systemic administration of MSG (1000 mg/kg, i.p.) induces dose- and time-dependent nocifensive- and spontaneous headache-like behaviour that was associated with a significant increase in peripheral CGRP levels^7^. This supports the theory (Fig. 3 of reference^1^) that elevated blood glutamate concentrations initiate headaches by directly sensitizing dural afferent fibers, and indirectly through CGRP (and possibly substance P) release from dural afferent endings to induce vasodilation^1,19,20^. This effect is a peripheral phenomenon as MSG administration does not significantly elevate CNS glutamate levels^21^. Previous research indicates that APV attenuates activation of trigeminovascular neurons by systemically administered MSG^6^. The current study provides additional evidence that the analgesic effect of APV is peripheral, as no evidence of motor dysfunction or altered coordination was uncovered with the dose employed. As blood glutamate levels are also elevated during and after a migraine headache, this finding suggests that peripheral NMDA receptors could be a potential target for novel migraine abortive drugs^3,4^.

Consistent with our previous findings, we found that systemic administration of MSG (1000 mg/kg, i.p.) induces nausea-like behaviour in a sexually dimorphic manner such that male rats display longer lying-on-belly behaviour than females rats^7^. Oral administration of MSG to healthy human subjects results in consistent reports of nausea^20^. This nausea may be induced through activation of gastric vagal afferents and/or the ability of glutamate to excite area postrema (chemoreceptor trigger zone) neurons^22^. Mechanoreceptive gastric vagal afferent fibers increase their firing rate in response to glutamate^23^. This may lead to a gastric distension-like sensation that results in the appearance of LOB behaviour, as an attempt by the rats to modulate it. Gastric distension associated excitation of vagal afferents is mediated by non-NMDA receptors, as previous work demonstrated its inhibition by the AMPA/kainate receptor antagonist CNQX, but not by APV^24^. Consistent with a lack of effect of APV on vagal afferent fibers, we found that APV had little effect on MSG-induced LOB behaviour. However, KYNA, which is a mixed excitatory amino acid receptor antagonist, appeared to slightly increase LOB behaviour. The increase in vagal afferent discharge induced by glutamate has been found to be inhibited by the 5-HT3 receptor antagonist granisetron, which suggests it may occur secondary to a local elevation of serotonin rather than by a direct action of glutamate on the vagal afferent fibers^25^.

The antiemetic agents MTC and OND, both of which have 5-HT3 antagonist activity, did exert an inhibitory effect on MSG-induced LOB behaviour. Both MTC and OND have central nervous system actions, and thus may also act in the area postrema or elsewhere in the central nausea generator to decrease nausea. Interestingly, we also found that olcegepant was also effective in inhibiting LOB behaviour. A recent systemic review and meta-analysis found strong evidence for the anti-nausea efficacy of gepants in episodic migraine^26^. Thus, the nausea behaviour induced by MSG administration responds to the same drugs that are found to be effective for the treatment of nausea in migraine. In this study we found that both MTC and OND were also effective in attenuating spontaneous headache-like behaviour. While MTC is used to abort migraines^27^, OND, though it has been shown to be effective in certain other types of headaches, is not usually used for migraine^28^. However, activation of 5-HT3 receptors has been shown to excite both dural and cranial muscle afferent fibers^29^. Thus, we speculate that part of the mechanism by which MSG produces headache-like behaviour in rats may result from increased peripheral serotonergic tone.

We note that one limitation of our studies was that the dose of MSG (1000 mg/kg) used to produce headache and nausea behaviours in rats is greater than that reported to induce headache and nausea in humans (150 mg/kg)^2^. Nevertheless, far greater doses of the CGRP receptor antagonists olcegepant and antiemetic drugs MTC and OND were also required to attenuate these behaviours in rats compared to humans, thus indicating that this model is potentially translatable to humans. Future research is required to address these limitations. A considerable variability in the magnitude of behavioural response to MSG was also noted when different groups of rats in this study were compared. We speculate that this variability is explained by individual differences in the rat’s responses to MSG. A similar variability in response to MSG ingestion by healthy humans is observed, where only between a third and a half report nausea and headache, respectively^20^.

Despite advances in migraine therapies, many people with migraine do not attain adequate relief or are resistant to available treatments^30^. There is, therefore, a need for additional prophylactic and abortive migraine therapies. Clinical evidence suggests a relationship between elevated plasma glutamate levels and migraine headache^1,3^. Ketamine and memantine, which are NMDA receptor antagonist drugs, have been investigated for migraine treatment and prophylaxis, respectively^31–33^. Several studies have also indicated that ketamine and AMPA receptor (LY293558; BGG492) antagonists were effective as abortive therapies in migraine with aura or familial hemiplegic migraine^31,34–36^. Despite their clinical efficacy, centrally mediated adverse side-effects have limited wide-scale use of these glutamate receptor antagonists for migraine pharmacotherapy^37^. The findings in our study suggest that peripherally restricted NMDA receptor antagonists may offer an alternative pathway for the development of prophylactic and/or abortive therapies.

The present study indicates that inhibition of peripheral NMDA receptors attenuates nocifensive- and headache-related behaviours produced by systemic administration of MSG in rats. MSG-induced nausea behaviour does not appear to be mediated by peripheral glutamate receptors but is sensitive to established antiemetic agents. Altogether, these findings further validate the MSG model of migraine-like headache and identify peripheral NMDA receptors as a future drug target.

## Methods

### Animals

Male (15) and Female (9) *Sprague Dawley* rats (weight 250–275 g at start of experiment) were used for these experiments. Rats were procured from Charles River laboratory and housed in groups of 2–3, under a light–dark cycle of light at 7:00 h to dark at 20:00 h in a temperature-controlled environment (20–25 °C) with free access to food and water. Experiments were specifically designed to minimize the number of rats used. For behavioural experiments, rats were acclimatized to the experimental set up and procedures for a minimum of 5 days prior to initiation of experiments. Rats were considered appropriately acclimatized when stable baseline mechanical thresholds (MTs) were obtained from stimulation of the temporalis muscle region (as described below). Rats were weighed daily. All animal experiments were approved by the Animal Care Committee of the University of British Columbia (A19-0174) and adhered to the ARRIVE guidelines 2.0. All methods were carried out in accordance with the guidelines and regulations of the Canadian Council on Animal Care.

### Drugs and Administration

Seven different compounds were used for these studies: The monohydrated monosodium salt of L-glutamic acid (MSG), the mixed glutamate receptor antagonist KYNA (sodium salt;), the selective NMDA receptor antagonist DL-2-amino-5-phosphonopentanoic acid (sodium salt; APV), the selective CGRP receptor antagonist olcegepant hydrochloride, the dopamine antagonist metoclopramide hydrochloride (MTC) and the serotonin (5HT) 3 receptor antagonist ondansetron hydrochloride (OND). MSG (Sigma-Aldrich Life Sciences, St. Louis, MO, USA) was dissolved in normal saline to make a 2 M stock solution. KYNA (Abcam Inc., Toronto, CA; 100 mg/mL), APV (Abcam Inc., Toronto, CA; 50 mg/mL), Olcegepant (MedChemExpress, NJ, USA; 1 mg/ml), MTC (Sigma-Aldrich Life Sciences, St. Louis, MO, USA; 5 mg/ml) and OND (Sigma-Aldrich Life Sciences, St. Louis, MO, USA; 1 mg/ml) were all dissolved in normal saline. All solutions were filtered using 0.22 µm Millex filter unit (Sigma-Aldrich Life Sciences, St. Louis, MO, USA) for sterility. Injections were then made from the filtrate and administered intraperitoneally (i.p.) in 2 mL volumes. All drugs were prepared freshly for each experiment and their administration randomized.

### Behavioural assays

#### Assessment of nocifensive, headache-like and nausea behaviours

Before each experiment, rats were allowed to acclimate to the testing area for 30 min. Rats were individually video recorded for 10 min before treatment (pre-injection video) and for a total of 1 h after treatment (post-injection video). The resultant mp4 video recording files were assigned to two blinded assessors for quantification of headache-like and nausea behaviours. Four distinct categories of non-evoked behaviour were assessed as previously described:

1. Nocifensive-like behaviour: Grimace score^38,39^
2. Headache-like behaviour: Head-flick (characterized as stereotypic rapid and arrhythmic vertical twitching of the head)^40^
3. Normal exploratory and grooming behaviours: Rearing (front paws lifted from the ground and placed on walls of the chambers), head scratches (movement patterns where the forehead was itched using the front or hind paws) and facial grooming (movement patterns where the facial areas are touched using the front or rear paws)
4. Nausea behaviour: Lying on belly (LOB; pressing abdomen-area between the fore paws and hind paws section onto the floor)^41,42^. Lying-on-belly behaviour is produced by the known nausea-inducing agent lithium chloride, and responds to treatment with the antiemetic ondansetron (OND)^41,42^.

The rat grimace score was assessed from frames captured at 10 min in the baseline video, and then at 10, 20, 30, 40, 50 and 60 min in the post-injection videos^38,39^. Frames were not captured when the rat was sleeping, grooming and or sniffing. If no clear photo could be captured during any period, then these time points were omitted from analysis. From the captured frames an unobstructed view of the face was cropped to remove body position.

The number of head-flick events was assessed for each 10-min epoch. The total duration of the exploratory, grooming and LOB behaviours in seconds was also assessed for each 10-min epoch. Exploratory and grooming behaviours post-injection (P1 = 0–10 min, P2 = 10–20 min, P3 = 20–30 min, P4 = 30–40 min, P5 = 40–50 min and P6 = 50–60 min) were normalized to baseline (B = 0–10 min) activity. Where the values for all behaviours scored by the two assessors, were significantly different, a consensus value was agreed upon for use in the final analysis (Intraclass Correlation Coefficient; r = 0.83; *p* = 0.008).

#### Mechanical withdrawal threshold (MT)

The MT of the temporalis muscle region was assessed using a rigid electronic von Frey hair (IITC Life Sciences, Woodland Hills, CA, USA) to evoke a withdrawal response as previously described^7,43^. At each time point, MT was obtained from the average of five successive mechanical stimulations alternating between the left and right temporalis muscle region. Post-injection MT was obtained every 10 min post i.p. injection and the relative MT was then calculated by dividing each post-injection MT by the baseline MT. The experimenter conducting the withdrawal assessments was blinded to the identity and order of the treatments.

### Rotarod assessment

In a separate group of *Sprague Dawley* rats (3 males and 3 females), we assessed for motor function deficits after systemic administration vehicle (normal saline), APV (50 mg/kg; i.p.), KYNA (100 mg/kg; i.p.) or olcegepant (1 mg/kg; i.p.) by employing the accelerated rotarod protocol (4–40 RPM)^44^. Rats were trained on the rotarod (IITC Life Sciences, Woodland Hills, CA, USA) every second day for a minimum of 6 days (3 sessions) until they were able to stay on the rod rotating at 40 RPM for at least 300 s for a total of three trials separated by 15 min intertrial intervals.

On experimental days, baseline latency was recorded as described above. Rats were then placed again on the rod (at, Max: 300 s) and the post-injection latency recorded at 5-, 20- and 35-min post-injection. Subjects that fell from the rod upon placement, but prior to pressing the start button, were given a “0” score for that trial (0 s latency to fall) and returned to their cage until their next consecutive trial. Rats that maintain their balance on the rotarod for the maximum time of 300 s were removed and placed into their cage until the next consecutive trial. Trials were separated by 48 h intervals.

### Experimental design

A transparent plexiglass chamber (8” × 8” × 8”) was equipped with mirrors behind the left, right and posterior walls of the chamber and a digital video recording camera placed 10 inches away from the anterior wall. The chamber was large enough to allow rats to roam freely and unhindered. Rats were acclimatized to the chamber by individually placing the rats into the chamber for 30 min each day for five days prior to experimental days. Experiments were conducted between 9:00 and 17:00 each day. The chamber was thoroughly cleaned with 70% isopropyl alcohol in between each rat. Each rat was given a 48-h rest period in between experiments. Previous work in healthy humans who were administered MSG 150 mg/kg daily for 5 days found no significant change in pain ratings or temporalis mechanical sensitivity at the end of 5 days compared to the first day^20^. Thus, given the apparent half-life of glutamate (30-min), we determined that a dosing interval of every 48-h would minimize the risk of sensitization to MSG^7,45^.

#### Glutamate and CGRP receptor antagonists

To investigate the effects of glutamate and CGRP receptor antagonists on MSG induced nocifensive, headache-like and nausea behaviours, 6 male and 6 female rats were individually placed in the chamber and video recorded for 10 min (baseline recording) and then given an i.p. injection of either MSG 1000 mg/kg alone or MSG 1000 mg/kg in combination with either KYNA (10, 50 or 100 mg/kg), APV (50 mg/kg) or olcegepant (1 mg/kg)^6,11,46^. Immediately after injection, the rat was placed into the chamber and video recorded for 1-h (post-injection recording). MT were also taken at baseline and every 10 min post-injection as described above. Each rat was tested every 48-h until it received all treatments in the protocol. The experimenter and assessors were blinded to the identity and order of the treatments.

#### Serotonin and dopamine receptor antagonists

To investigate the effects of serotonin and dopamine receptor antagonists on MSG induced nausea- and headache-like behaviours, a separate cohort of 6 male rats using the same experimental paradigm described in Study 1 was used. Male, but not female rats were examined in these experiments, as male rats have been previously shown to have the most robust nausea behavior in response to MSG administration^7^. Rats were individually placed in the chamber and video recorded for 10 min (baseline recording) and then given an i.p. injection of either MSG 1000 mg/kg alone or MSG 1000 mg/kg in combination with either MTC (3 mg/kg) or OND (0.5 mg/kg). Immediately after injection, the rat was placed into the chamber and video recorded for 1-h (post-injection recording). MTs were also taken at baseline and every 10 min post-injection as described above. Each rat was tested every 48-h until it received all treatments in the protocol. The experimenter and assessors were blinded to the identity and order of the treatments.

### Data and statistical analysis

Sample size was based on a previous study where significant effects of MSG administration on behaviours were demonstrated in groups of 6 rats^7^. Statistical analysis was performed using the GraphPad Prism (Version 9.1.1). Normality was assessed using the Shapiro–Wilk normality test. Where data was non-normally distributed, data transformation by square root (SQRT) was used to achieve normally distributed data with equal variances. Data were analysed using repeated measures two-way mixed model ANOVAs with post-hoc Dunnett’s tests (time and treatment as factors). Sex-related differences were analysed by repeated measures two-way mixed model ANOVA with post-hoc Dunnett’s test (sex and time as factors). For all analyses, *p* < 0.05 was considered statistically significant. Data in the text are reported as mean ± SEM (Standard Error of the Mean).

## Supplementary Information

Supplementary Information.

## Data Availability

The data that support the findings of this study are available from the corresponding author, BEC, upon reasonable request.

## Abbreviations

5HT: 5-Hydroxytryptamine
AMPA: α-Amino-3-hydroxy-5-methyl-5-isoxazolepropionate
ANOVA: Analysis of variance
APV: (2R)-amino-5-phosphonovaleric acid
CGRP: Calcitonin-gene related peptide
CNQX: Cyanquixaline (6-cyano-7-nitroquinoxaline-2,3-dione)
GTN: Glycerol trinitrate (nitroglycerin)
IP: Intraperitoneally
KYNA: Kynurenic acid
MSG: Monosodium glutamate
MT: Mechanical threshold
MTC: Metoclopramide
NMDA: *N*-Methyl-d-aspartate
OND: Ondansetron
RGS: Rat grimace score
RPM: Revolutions per minute
