# Supplementary material for: Peripheral N-methyl-d-aspartate receptor activation contributes to monosodium glutamate-induced headache but not nausea behaviours in rats
Source: Sci Rep. 2022 Aug 16;12:13894. doi: 10.1038/s41598-022-18290-w (PMC9381496; doi:10.1038/s41598-022-18290-w)
Supplement: Supplementary file 1 — Supplementary Information. [file 41598_2022_18290_MOESM1_ESM.pdf]

Peripheral N-Methyl-D-Aspartate Receptor Activation Contributes to Monosodium Glutamate-Induced Headache but not Nausea Behaviours in Rats

Supplementary Information File

Tarique Benbow[1], Felisha Teja[1], Afrooz Sheikhi[1], Fernando G. Exposto[2], Peter Svensson[2] and Brian E. Cairns[1*]

[1]Faculty of Pharmaceutical Sciences, University of British Columbia, 2405 Wesbrook Mall, Vancouver, Canada V6T 1Z3

[2]Section for Orofacial Pain and Jaw Function, Department of Dentistry and Oral Health, Aarhus University, Vennelyst Boulevard 9, 8000 Aarhus C, Denmark

Address for correspondence:
Brian E Cairns, PhD, DrMed, ACPR
Professor
Faculty of Pharmaceutical Sciences
University of British Columbia
2405 Wesbrook Mall, Vancouver, Canada, V6T 1Z3
Email: brian.cairns@ubc.ca

Data Generated and Analyzed

***Study 1** – Glutamate and CGRP Receptor Antagonists*

***Study 2** – Rotarod assessment*

***Study 3** – Serotonin and Dopamine Receptor Antagonists*

***Sex Differences** – Statistical Results*

Facial Grooming - Study 1

	MSG + Vehicle	MSG + Vehicle	MSG + Vehicle	MSG + Vehicle	MSG + Vehicle	MSG + Vehicle	MSG + Vehicle	MSG + Vehicle	MSG + Vehicle	MSG + Vehicle	MSG + Vehicle	MSG + Vehicle
	M1	M2	M3	M4	M5	M6	F1	F2	F3	F4	F5	F6
P1	0.265	0	0	0	1.393	0	0.37	0.116	0	0	0	0.646
P2	0.311	0	0	0.062	0.429	0.319	0	0	0	0	0.85	0.754
P3	0	0	0	0	0.5	0	0.102	0.28	1.915	0.716	1.001	0.231
P4	0.195	0	0	0.479	0.571	0.511	0	1.302	0	0.087	0	1.246
P5	0.448	1.035	0	0	0.429	0	0.098	0	0	1.074	0.671	0
P6	0	0	6.883	0.955	0	0	0	0	0.737	0	0.877	0.215

	MSG + APV 50 mg/kg	MSG + APV 50 mg/kg	MSG + APV 50 mg/kg	MSG + APV 50 mg/kg	MSG + APV 50 mg/kg	MSG + APV 50 mg/kg	MSG + APV 50 mg/kg	MSG + APV 50 mg/kg	MSG + APV 50 mg/kg	MSG + APV 50 mg/kg	MSG + APV 50 mg/kg	MSG + APV 50 mg/kg
	M1	M2	M3	M4	M5	M6	F1	F2	F3	F4	F5	F6
P1	0.003	0.004	1.303	0.01	0.14	0.073	0	2.156	0	0.344	0.569	7.429
P2	0.08	0	0.763	0.519	1.5	0	0	0.405	0	0.144	0.569	1.993
P3	0	1.18	0.791	0.192	2	0	0	0	0	0.411	1.128	2.721
P4	0	0	0	1.731	1.611	0.354	0	0	0	1.49	0.504	0.671
P5	1.419	0	0.58	0.365	3.111	0	0	0.385	0.992	0.911	0	1.514
P6	0	1.245	0.311	0.385	1.056	0.898	0	0	0	0.678	1.028	0.671

	MSG + KYNA 10 mg/kg	MSG + KYNA 10 mg/kg	MSG + KYNA 10 mg/kg	MSG + KYNA 10 mg/kg	MSG + KYNA 10 mg/kg	MSG + KYNA 10 mg/kg	MSG + KYNA 10 mg/kg	MSG + KYNA 10 mg/kg	MSG + KYNA 10 mg/kg	MSG + KYNA 10 mg/kg	MSG + KYNA 10 mg/kg	MSG + KYNA 10 mg/kg
	M1	M2	M3	M4	M5	M6	F1	F2	F3	F4	F5	F6
P1	1.18	0	0	0	0.36	0	4.211	0	0	0	1.11	0
P2	0.872	0	0	0.359	0	0	0	0	0	0	1.505	0.777
P3	0.726	0.854	0	0	1.44	0	0	2.252	0	1.002	0	0.859
P4	0.308	0.528	0.445	0	0	0	0.683	0	0	1.559	0.835	2.012
P5	0.6	1.31	0	0.944	2.115	0.106	2.117	0.701	0.612	0.956	0.541	1.452
P6	0	0	0	0.09	0.81	0	0	1.452	0	0	0.908	1.481

	MSG + KYNA 50 mg/kg	MSG + KYNA 50 mg/kg	MSG + KYNA 50 mg/kg	MSG + KYNA 50 mg/kg	MSG + KYNA 50 mg/kg	MSG + KYNA 50 mg/kg	MSG + KYNA 50 mg/kg	MSG + KYNA 50 mg/kg	MSG + KYNA 50 mg/kg	MSG + KYNA 50 mg/kg	MSG + KYNA 50 mg/kg	MSG + KYNA 50 mg/kg
	M1	M2	M3	M4	M5	M6	F1	F2	F3	F4	F5	F6
P1	0	0	0	0.426	0	0	0	0	0.334	0	0	0
P2	0.054	0	0.013	0.238	1.5	0	0.599	0	0.012	0	0.58	0.405
P3	0.263	0.275	0	0.693	2	0	0.099	0	0.08	1.079	0.074	0.434
P4	0	0.435	0	0	1.611	0	0	0.802	0.104	0.259	0	0.841
P5	0	0.757	0.38	0	3.111	0	0	0.677	0	0.741	0	0.536
P6	0.263	0	0.98	0	1.056	1.934	0	0	0	0	0.136	0.855

	MSG + KYNA 100 mg/kg	MSG + KYNA 100 mg/kg	MSG + KYNA 100 mg/kg	MSG + KYNA 100 mg/kg	MSG + KYNA 100 mg/kg	MSG + KYNA 100 mg/kg	MSG + KYNA 100 mg/kg	MSG + KYNA 100 mg/kg	MSG + KYNA 100 mg/kg	MSG + KYNA 100 mg/kg	MSG + KYNA 100 mg/kg	MSG + KYNA 100 mg/kg
	M1	M2	M3	M4	M5	M6	F1	F2	F3	F4	F5	F6
P1	0	0	0	0	0.219	0	0.688	0	0.306	0	0	0
P2	0	0	0.358	0	0.875	0	0	0	0	0	0	0.295
P3	0.902	0	0	0	0.938	0	0.904	0.603	0.143	0	0.764	0.228
P4	0.111	0.432	0	0	0.375	0	0	2.055	0.102	0.059	0.514	0
P5	1.146	0	1.802	0	0.75	0.337	0	0	0	0	0.903	0
P6	1.012	0	1.339	0	0	0	0.214	1.024	0.45	0.201	0.722	0

	MSG + Olcegepant 1 mg/kg	+ Olcegepant 1 mg/kg	+ Olcegepant 1 mg/kg	+ Olcegepant 1 mg/kg	+ Olcegepant 1 mg/kg	+ Olcegepant 1 mg/kg	+ Olcegepant 1 mg/kg	+ Olcegepant 1 mg/kg	+ Olcegepant 1 mg/kg	+ Olcegepant 1 mg/kg	+ Olcegepant 1 mg/kg	+ Olcegepant 1 mg/kg
	M1	M2	M3	M4	M5	M6	F1	F2	F3	F4	F5	F6
P1	0	0	0	0	0.5	0	0	0	1.889	0	0	0
P2	0.338	0	0.603	0	0	0	0.224	0.58	0.528	0.162	0.639	0.757
P3	0	0.609	0	0.181	2.273	0.262	0.408	0.913	0	0.162	0.236	0.953
P4	0.352	0.882	1.192	0	1.045	0.373	0	0.703	0.083	0.011	0	1.082
P5	0	0	1.027	0	0.773	0	0.25	0	0	0.248	0	0
P6	0	0.628	1.37	0.434	1.182	0.437	0	0	0.639	0.517	0.674	0.888

Rat Grimace Score - Study 1

	Time (min)	MSG + Vehicle	MSG + Vehicle	MSG + Vehicle	MSG + Vehicle	MSG + Vehicle	MSG + Vehicle	MSG + Vehicle	MSG + Vehicle	MSG + Vehicle	MSG + Vehicle	MSG + Vehicle	MSG + Vehicle
		F1	F2	F3	F4	F5	F6	M1	M2	M3	M4	M5	M6
P1	10	2	2	1.75	2	2	2	1	2	2	1.5	1.75	2
P2	20	2	2	2	2	2	1	0.75	1.75	2	1.25	1.25	1.25
P3	30	1.25	2	2	1	1.75	0.5	0.25	1	2	1.5	0.75	0.75
P4	40	0	1.75	2	1.75	2	1	1	1.25	2	0	0.5	0.5
P5	50	0	1	2	0	1	0	0	0.75	1.5	0	0	0
P6	60	0	1.75	2	0	2	0	0	0	0	0	0	0

	Time (min)	MSG + APV 50 mg/kg	MSG + APV 50 mg/kg	MSG + APV 50 mg/kg	MSG + APV 50 mg/kg	MSG + APV 50 mg/kg	MSG + APV 50 mg/kg	MSG + APV 50 mg/kg	MSG + APV 50 mg/kg	MSG + APV 50 mg/kg	MSG + APV 50 mg/kg	MSG + APV 50 mg/kg	MSG + APV 50 mg/kg
		F1	F2	F3	F4	F5	F6	M1	M2	M3	M4	M5	M6
P1	10	1	0	0	1.5	0	0	1.5	0	0	0.75	2	0
P2	20	2	0	2	0.75	0	0	0.25	2	0	0.25	2	0
P3	30	2	0	2	0	0	0	0	1	0	0	0.25	0
P4	40	1	0.5	2	0	0	0.25	0	0.75	0	0	0	0
P5	50	2	0	1.25	0	0.25	0	0	0.25	0	0	0	0
P6	60	1.75	0	0.75	0	0	0.25	0.25	0	0	0	0	0

	Time (min)	MSG + KYNA 10 mg/kg	MSG + KYNA 10 mg/kg	MSG + KYNA 10 mg/kg	MSG + KYNA 10 mg/kg	MSG + KYNA 10 mg/kg	MSG + KYNA 10 mg/kg	MSG + KYNA 10 mg/kg	MSG + KYNA 10 mg/kg	MSG + KYNA 10 mg/kg	MSG + KYNA 10 mg/kg	MSG + KYNA 10 mg/kg	MSG + KYNA 10 mg/kg
		F1	F2	F3	F4	F5	F6	M1	M2	M3	M4	M5	M6
P1	10	0	2	2	2	0.25	1	0.25	0	1.75	2	0.75	0
P2	20	0.25	2	2	2	0	0.75	0.25	0.25	1.75	2	1	0
P3	30	0.5	2	2	1.5	0	0	0	0.5	1	1.5	0.75	0
P4	40	0	1.75	1.5	1	0	0	0	0.5	0.25	0.5	0.25	0
P5	50	0.25	0	1.25	1	0	0	0	0	0	0	0	0
P6	60	0.5	0	1.5	0.75	0	0	0	0	0	0	0	0

	Time (min)	MSG + KYNA 50 mg/kg	MSG + KYNA 50 mg/kg	MSG + KYNA 50 mg/kg	MSG + KYNA 50 mg/kg	MSG + KYNA 50 mg/kg	MSG + KYNA 50 mg/kg	MSG + KYNA 50 mg/kg	MSG + KYNA 50 mg/kg	MSG + KYNA 50 mg/kg	MSG + KYNA 50 mg/kg	MSG + KYNA 50 mg/kg	MSG + KYNA 50 mg/kg
		F1	F2	F3	F4	F5	F6	M1	M2	M3	M4	M5	M6
P1	10	1.75	1.5	1.25	0	0	0	2	0	2	0	0	2
P2	20	1.25	1.5	0.25	2	0	0	2	1.75	2	0	0.25	2
P3	30	1	2	1.25	0.75	0	0	2	1	2	0	0.5	1.25
P4	40	1	1.25	0.75	1	0	1	1	1.25	1	0	0	2
P5	50	0	0.75	0	0.5	0.25	0	1.25	0.75	0.25	0	0	0
P6	60	0	0	0	0.25	0	0	0	0	0	0	0	0

	Time (min)	MSG + KYNA 100 mg/kg	MSG + KYNA 100 mg/kg	MSG + KYNA 100 mg/kg	MSG + KYNA 100 mg/kg	MSG + KYNA 100 mg/kg	MSG + KYNA 100 mg/kg	MSG + KYNA 100 mg/kg	MSG + KYNA 100 mg/kg	MSG + KYNA 100 mg/kg	MSG + KYNA 100 mg/kg	MSG + KYNA 100 mg/kg	MSG + KYNA 100 mg/kg
		F1	F2	F3	F4	F5	F6	M1	M2	M3	M4	M5	M6
P1	10	1	2	0	1	1	2	0	1	2	0.5	2	1.75
P2	20	2	2	0	0	1	1.75	2	0.75	1	0.75	2	2
P3	30	0.25	2	0	0	1	2	2	0.75	1	0	0.75	1.5
P4	40	0.5	1	0	1.75	0	2	1.75	1	0.75	1	1	1.75
P5	50	1	1.75	0	1.25	0	1.25	1.5	0	0	0.5	0.25	0
P6	60	0	0	0	0	0	2	2	0	0	0.5	0	0

	Time (min)	+ Olcegepant 1 mg/kg	+ Olcegepant 1 mg/kg	+ Olcegepant 1 mg/kg	+ Olcegepant 1 mg/kg	+ Olcegepant 1 mg/kg	+ Olcegepant 1 mg/kg	+ Olcegepant 1 mg/kg	+ Olcegepant 1 mg/kg	+ Olcegepant 1 mg/kg	+ Olcegepant 1 mg/kg	+ Olcegepant 1 mg/kg	+ Olcegepant 1 mg/kg
		F1	F2	F3	F4	F5	F6	M1	M2	M3	M4	M5	M6
P1	10	1.75	0.75	1.25	0	1.5	0.75	1.5	0.75	1	1.25	1.25	2
P2	20	1.75	1	0	2	1.25	1	1.75	0.75	1	1	1.25	2
P3	30	1.25	0.75	0	1.25	2	1.5	0.75	0	0.5	0.75	1.25	1.25
P4	40	0	0	0	2	1.5	0.5	1.75	0	0	0.75	0.75	0.25
P5	50	0	1	0	0	0	0.25	0.5	0	0	0.25	0.5	0.25
P6	60	0	0.25	0	0	0	0	0	0	0	0	0	0

Head Flicks - Study 1

	Time (min)	MSG + Vehicle	MSG + Vehicle	MSG + Vehicle	MSG + Vehicle	MSG + Vehicle	MSG + Vehicle	MSG + Vehicle	MSG + Vehicle	MSG + Vehicle	MSG + Vehicle	MSG + Vehicle	MSG + Vehicle
		F1	F2	F3	F4	F5	F6	M1	M2	M3	M4	M5	M6
P1	10	1	2	3	8	3	5	1	4	5	7	1	4
P2	20	1	1	1	4	1	2	1	2	3	5	1	1
P3	30	0	0	0	0	1	0	1	2	0	1	0	0
P4	40	0	1	0	0	1	2	0	0	0	0	1	0
P5	50	0	0	0	0	0	2	0	0	0	0	0	0
P6	60	0	0	1	0	0	2	0	0	0	0	0	1

	Time (min)	MSG + APV 50 mg/kg	MSG + APV 50 mg/kg	MSG + APV 50 mg/kg	MSG + APV 50 mg/kg	MSG + APV 50 mg/kg	MSG + APV 50 mg/kg	MSG + APV 50 mg/kg	MSG + APV 50 mg/kg	MSG + APV 50 mg/kg	MSG + APV 50 mg/kg	MSG + APV 50 mg/kg	MSG + APV 50 mg/kg
		F1	F2	F3	F4	F5	F6	M1	M2	M3	M4	M5	M6
P1	10	2	0	2	2	2	0	1	2	1	2	1	2
P2	20	1	0.5	1	0	0	0.5	0	0	0	0	3	0
P3	30	1.5	0	0.5	1	0	0	0	0	0	0	0	1
P4	40	0	0	0	3	0	0	1	0	0	0	0	0
P5	50	1	0	1	1	1	1	0	0	1	0	3	1
P6	60	0	0	0	1	1	0	0	0	0.5	0	0	0

	Time (min)	MSG + KYNA 10 mg/kg	MSG + KYNA 10 mg/kg	MSG + KYNA 10 mg/kg	MSG + KYNA 10 mg/kg	MSG + KYNA 10 mg/kg	MSG + KYNA 10 mg/kg	MSG + KYNA 10 mg/kg	MSG + KYNA 10 mg/kg	MSG + KYNA 10 mg/kg	MSG + KYNA 10 mg/kg	MSG + KYNA 10 mg/kg	MSG + KYNA 10 mg/kg
		F1	F2	F3	F4	F5	F6	M1	M2	M3	M4	M5	M6
P1	10	0	1	5	2	0	2	0	2	1	10	3	2
P2	20	0	0	1.5	0	0	1	1	1	0	0	0	2
P3	30	0	0	0.5	0	0	1	0	1	0	0	0	1
P4	40	0	1	0	0	0	1	1	1	0	0	0	0
P5	50	0.5	0	0	0	0	1	0	0	0	1	1	0
P6	60	0	0	0	1	0	0	0	0	0	0	0	0

	Time (min)	MSG + KYNA 50 mg/kg	MSG + KYNA 50 mg/kg	MSG + KYNA 50 mg/kg	MSG + KYNA 50 mg/kg	MSG + KYNA 50 mg/kg	MSG + KYNA 50 mg/kg	MSG + KYNA 50 mg/kg	MSG + KYNA 50 mg/kg	MSG + KYNA 50 mg/kg	MSG + KYNA 50 mg/kg	MSG + KYNA 50 mg/kg	MSG + KYNA 50 mg/kg
		F1	F2	F3	F4	F5	F6	M1	M2	M3	M4	M5	M6
P1	10	2	2.5	0	3	2.5	2	3	3	0	0	0	2
P2	20	2	1.5	0	0	4	1	1	0	0	0	0	0
P3	30	0.5	2	0	1	0	1	2	0	0	0	3	1
P4	40	2	0	0	1	0	1	0	0	0	0	0	0
P5	50	1	0	0	0	0	1	1	0	0	0	0	1
P6	60	0	0	0	1	1	0	0	0	1	0	0	0

	Time (min)	MSG + KYNA 100 mg/kg	MSG + KYNA 100 mg/kg	MSG + KYNA 100 mg/kg	MSG + KYNA 100 mg/kg	MSG + KYNA 100 mg/kg	MSG + KYNA 100 mg/kg	MSG + KYNA 100 mg/kg	MSG + KYNA 100 mg/kg	MSG + KYNA 100 mg/kg	MSG + KYNA 100 mg/kg	MSG + KYNA 100 mg/kg	MSG + KYNA 100 mg/kg
		F1	F2	F3	F4	F5	F6	M1	M2	M3	M4	M5	M6
P1	10	0	1.5	0	2	1	2.5	9	3	1	10	2	4
P2	20	0	1.5	0	0	0	1.5	0	0	0	0	1	0
P3	30	0	0.5	0	0	0	1	0	0	0	0	0	1
P4	40	1	0.5	0	1	0	1	0	1	0	0	1	2
P5	50	0	0	0	0	1	0	2	0	0	1	0	3
P6	60	1	0.5	2	0	0	0	0	0	0	0	2	4

	Time (min)	Olcegepant 10 mg/kg	Olcegepant 10 mg/kg	Olcegepant 10 mg/kg	Olcegepant 10 mg/kg	Olcegepant 10 mg/kg	Olcegepant 10 mg/kg	Olcegepant 10 mg/kg	Olcegepant 10 mg/kg	Olcegepant 10 mg/kg	Olcegepant 10 mg/kg	Olcegepant 10 mg/kg	Olcegepant 10 mg/kg
		F1	F2	F3	F4	F5	F6	M1	M2	M3	M4	M5	M6
P1	10	1	1	0	0	0	3	2	0	2	2	2	2
P2	20	0	1	3	1	0	0	0	0	0	1	0	0
P3	30	0	1	1	0	0	0	0	1	0	0	2	1
P4	40	0	0	2	0	0	0	1	0	0	0	0	0
P5	50	0	0	0	2	0	0	0	0	0	0	0	0
P6	60	0	0	0	0	0	0	0	0	0	0	0	0

Head Scratches - Study 1

	Time (min)	MSG + Vehicle	MSG + Vehicle	MSG + Vehicle	MSG + Vehicle	MSG + Vehicle	MSG + Vehicle	MSG + Vehicle	MSG + Vehicle	MSG + Vehicle	MSG + Vehicle	MSG + Vehicle	MSG + Vehicle
		F1	F2	F3	F4	F5	F6	M1	M2	M3	M4	M5	M6
P1	10	0.3	0	0	0	0	1.176	0.073	0	0	0	0.551	0
P2	20	0	0	0	0	0	1.422	0	0	0	0	0.102	0.087
P3	30	0	0	0	0.54	0.362	0.58	0	0	0	0	0.205	0
P4	40	0	0.866	0	0	0.082	0.843	0	0	0	0.133	0.348	0.333
P5	50	0	0	0	0.84	0.42	0	0.268	1.035	0	0	0.878	0
P6	60	0	0	0	0	0.754	0.088	0	0	13	1.709	0	0

	Time (min)	MSG + APV 50 mg/kg	MSG + APV 50 mg/kg	MSG + APV 50 mg/kg	MSG + APV 50 mg/kg	MSG + APV 50 mg/kg	MSG + APV 50 mg/kg	MSG + APV 50 mg/kg	MSG + APV 50 mg/kg	MSG + APV 50 mg/kg	MSG + APV 50 mg/kg	MSG + APV 50 mg/kg	MSG + APV 50 mg/kg
		F1	F2	F3	F4	F5	F6	M1	M2	M3	M4	M5	M6
P1	10	0.527	0.486	0	0.367	0.182	22	0.2	0.4	2.458	0.3	0.73	0.04
P2	20	0.129	0.371	0	1.608	0.409	17	0	0	0.792	0	0.603	0
P3	30	0.075	0.486	0	0.192	0.545	14.625	0.092	0.254	0.792	0.194	1.564	0
P4	40	0	0.086	0	4.208	0.091	1.625	0	0	1.146	0.531	0.683	0.073
P5	50	0	0.257	0.992	0.942	0	2.375	0.391	0	2.021	0.106	1.567	0
P6	60	0.011	0	0	0.45	1.523	0	0	0.305	1.083	0.206	1.122	0.276

	Time (min)	MSG + KYNA 10 mg/kg	MSG + KYNA 10 mg/kg	MSG + KYNA 10 mg/kg	MSG + KYNA 10 mg/kg	MSG + KYNA 10 mg/kg	MSG + KYNA 10 mg/kg	MSG + KYNA 10 mg/kg	MSG + KYNA 10 mg/kg	MSG + KYNA 10 mg/kg	MSG + KYNA 10 mg/kg	MSG + KYNA 10 mg/kg	MSG + KYNA 10 mg/kg
		F1	F2	F3	F4	F5	F6	M1	M2	M3	M4	M5	M6
P1	10	5.15	0	0	0	1.202	0	1.504	0	0	0	0.551	0
P2	20	0	0	0	0	3.854	0.456	0.772	0	0	0.035	0	0
P3	30	0	1.526	0	1.09	0.199	0.266	0.366	1	0	0	0.854	0
P4	40	0.675	0	0	5.174	0.869	1.368	0	1.292	0.019	0	0	0
P5	50	0.55	0.688	0.612	0.764	0.667	0.234	0.17	3.917	0	0.177	1.505	0
P6	60	0	0.624	0	0	1.315	0.077	0	0	0	0.188	0.596	0

	Time (min)	MSG + KYNA 50 mg/kg	MSG + KYNA 50 mg/kg	MSG + KYNA 50 mg/kg	MSG + KYNA 50 mg/kg	MSG + KYNA 50 mg/kg	MSG + KYNA 50 mg/kg	MSG + KYNA 50 mg/kg	MSG + KYNA 50 mg/kg	MSG + KYNA 50 mg/kg	MSG + KYNA 50 mg/kg	MSG + KYNA 50 mg/kg	MSG + KYNA 50 mg/kg
		F1	F2	F3	F4	F5	F6	M1	M2	M3	M4	M5	M6
P1	10	0	0	0.334	0	0	0	0	0	0	0.096	3	0
P2	20	0.044	0	0.012	0	0.236	0	0	0	0	0.098	8.25	0
P3	30	0.067	0	0.08	0.165	0.118	0.224	0.043	0.533	0	0.342	7	0
P4	40	0	0	0.104	0.519	0	0.633	0	0	0.017	0.17	3.25	0
P5	50	0	0.117	0	0.27	0	0.204	0	1.367	0	0.365	3.25	0
P6	60	0	0	0	0.125	0.236	1.102	0.029	0	0.45	0.024	7.25	0.274

	Time (min)	MSG + KYNA 100 mg/kg	MSG + KYNA 100 mg/kg	MSG + KYNA 100 mg/kg	MSG + KYNA 100 mg/kg	MSG + KYNA 100 mg/kg	MSG + KYNA 100 mg/kg	MSG + KYNA 100 mg/kg	MSG + KYNA 100 mg/kg	MSG + KYNA 100 mg/kg	MSG + KYNA 100 mg/kg	MSG + KYNA 100 mg/kg	MSG + KYNA 100 mg/kg
		F1	F2	F3	F4	F5	F6	M1	M2	M3	M4	M5	M6
P1	10	0.36	0	0.306	0	0	0	0	0	0	0	0.156	0
P2	20	0	0	0	0	0.026	0	0	0	0	0	2.061	0
P3	30	0.811	0	0.143	0	0	0	0.261	0	0	0	0.95	0
P4	40	0.171	0.836	0.102	0	0	0	0	0.2	0	0.283	3.117	0
P5	50	0.027	0	0	0	0.299	0	0.739	0	0.948	0	2.694	0.107
P6	60	0.28	0.925	0.45	0.218	0.3	0	0.211	0	0.83	0	0	0

	Time (min)	Olcegepant 10 mg/kg	Olcegepant 10 mg/kg	Olcegepant 10 mg/kg	Olcegepant 10 mg/kg	Olcegepant 10 mg/kg	Olcegepant 10 mg/kg	Olcegepant 10 mg/kg	Olcegepant 10 mg/kg	Olcegepant 10 mg/kg	Olcegepant 10 mg/kg	Olcegepant 10 mg/kg	Olcegepant 10 mg/kg
		F1	F2	F3	F4	F5	F6	M1	M2	M3	M4	M5	M6
P1	10	0	0	1.889	0	0.381	0	0	0	0	0	0.845	0
P2	20	0.028	0	0.528	0	0	0.158	0.035	0	0	0	0	0
P3	30	0.284	1.079	0	0.071	0.276	0.512	0	0	0	0	0.72	0
P4	40	0	0.617	0.083	0	0	2.33	0.123	0.083	0.189	0.633	0.153	0.208
P5	50	0.046	0	0	0	0	0	0	0	0.58	0	0.051	0
P6	60	0.037	0	0.639	0.339	0	0.089	0	1.396	0.648	0.742	0.258	0.368

Lying-on-Belly - Study 1

	MSG + Vehicle	MSG + Vehicle	MSG + Vehicle	MSG + Vehicle	MSG + Vehicle	MSG + Vehicle	MSG + Vehicle	MSG + Vehicle	MSG + Vehicle	MSG + Vehicle	MSG + Vehicle	MSG + Vehicle
	M1	M2	M3	M4	M5	M6	F1	F2	F3	F4	F5	F6
P1	0	385	249	334	324	379	0	0	339	0	0	0
P2	0	475.5	0	0	0	271.5	0	0	0	0	0	0
P3	266.5	94	0	0	0	36.5	0	0	0	0	0	85
P4	0	0	0	0	0	0	0	0	0	0	0	0
P5	0	0	0	0	0	0	0	0	0	0	0	0
P6	0	0	0	0	0	0	0	0	0	0	0	0

	MSG + APV 50 mg/kg	MSG + APV 50 mg/kg	MSG + APV 50 mg/kg	MSG + APV 50 mg/kg	MSG + APV 50 mg/kg	MSG + APV 50 mg/kg	MSG + APV 50 mg/kg	MSG + APV 50 mg/kg	MSG + APV 50 mg/kg	MSG + APV 50 mg/kg	MSG + APV 50 mg/kg	MSG + APV 50 mg/kg
	M1	M2	M3	M4	M5	M6	F1	F2	F3	F4	F5	F6
P1	0	179	0	161	0	390	303	0	419	0	0	0
P2	0	0	0	0	0	617	0	0	0	0	0	0
P3	0	0	0	0	0	155	0	0	0	0	0	0
P4	0	0	0	0	0	0	0	0	0	0	0	0
P5	0	0	0	0	0	0	0	0	0	0	0	0
P6	0	0	0	0	0	0	155	0	0	0	0	249

	MSG + KYNA 10 mg/kg	MSG + KYNA 10 mg/kg	MSG + KYNA 10 mg/kg	MSG + KYNA 10 mg/kg	MSG + KYNA 10 mg/kg	MSG + KYNA 10 mg/kg	MSG + KYNA 10 mg/kg	MSG + KYNA 10 mg/kg	MSG + KYNA 10 mg/kg	MSG + KYNA 10 mg/kg	MSG + KYNA 10 mg/kg	MSG + KYNA 10 mg/kg
	M1	M2	M3	M4	M5	M6	F1	F2	F3	F4	F5	F6
P1	0	290	126	378	342	407	0	0	373	116	0	154
P2	0	310	322	503	369	573	0	0	0	19	0	0
P3	0	0	250	0	251	535	0	0	0	0	0	0
P4	0	20	0	0	0	0	0	0	0	0	0	0
P5	0	0	0	0	0	0	0	0	0	0	0	0
P6	0	500	0	0	0	0	40	0	0	0	0	0

	MSG + KYNA 50 mg/kg	MSG + KYNA 50 mg/kg	MSG + KYNA 50 mg/kg	MSG + KYNA 50 mg/kg	MSG + KYNA 50 mg/kg	MSG + KYNA 50 mg/kg	MSG + KYNA 50 mg/kg	MSG + KYNA 50 mg/kg	MSG + KYNA 50 mg/kg	MSG + KYNA 50 mg/kg	MSG + KYNA 50 mg/kg	MSG + KYNA 50 mg/kg
	M1	M2	M3	M4	M5	M6	F1	F2	F3	F4	F5	F6
P1	420	258	0	0	90	327	267	0	0	331	255	91
P2	0	562	0	0	363	592	131	0	0	0	0	0
P3	0	0	0	0	0	476	0	0	0	0	0	0
P4	0	0	0	0	0	28	167	113	0	0	70	0
P5	0	0	0	0	0	8	493	0	0	0	0	0
P6	0	0	0	0	0	0	505	0	0	0	75	0

	MSG + KYNA 100 mg/kg	MSG + KYNA 100 mg/kg	MSG + KYNA 100 mg/kg	MSG + KYNA 100 mg/kg	MSG + KYNA 100 mg/kg	MSG + KYNA 100 mg/kg	MSG + KYNA 100 mg/kg	MSG + KYNA 100 mg/kg	MSG + KYNA 100 mg/kg	MSG + KYNA 100 mg/kg	MSG + KYNA 100 mg/kg	MSG + KYNA 100 mg/kg
	M1	M2	M3	M4	M5	M6	F1	F2	F3	F4	F5	F6
P1	358.5	392.5	153.5	378	318.5	290	0	0	0	0	0	222
P2	0	572	0	502.5	290	361.5	0	0	0	16.5	0	0
P3	0	0	0	0	100.5	381	0	0	0	0	0	0
P4	0	0	0	0	0	0	0	0	0	0	0	0
P5	0	0	0	0	136	0	0	0	0	0	0	0
P6	0	0	0	0	0	0	0	0	0	0	0	0

	MSG + Olcegepant 1 mg/kg	+ Olcegepant 1 mg/kg	+ Olcegepant 1 mg/kg	+ Olcegepant 1 mg/kg	+ Olcegepant 1 mg/kg	+ Olcegepant 1 mg/kg	+ Olcegepant 1 mg/kg	+ Olcegepant 1 mg/kg	+ Olcegepant 1 mg/kg	+ Olcegepant 1 mg/kg	+ Olcegepant 1 mg/kg	+ Olcegepant 1 mg/kg
	M1	M2	M3	M4	M5	M6	F1	F2	F3	F4	F5	F6
P1	0	0	0	0	0	125	0	0	0	0	0	0
P2	0	0	0	0	0	585	0	0	0	179	0	0
P3	0	0	0	415	124	206	0	0	0	177	0	0
P4	0	0	0	0	432	0	0	0	182	0	0	0
P5	0	0	0	0	0	0	0	0	0	0	0	0
P6	0	0	0	0	0	0	0	0	35	0	0	0

Lying-on-Belly (Female) - Study 1

	MSG + Vehicle	MSG + Vehicle	MSG + Vehicle	MSG + Vehicle	MSG + Vehicle	MSG + Vehicle
	F1	F2	F3	F4	F5	F6
P1	0	0	339	0	0	0
P2	0	0	0	0	0	0
P3	0	0	0	0	0	85
P4	0	0	0	0	0	0
P5	0	0	0	0	0	0
P6	0	0	0	0	0	0

	MSG + APV 50 mg/kg	MSG + APV 50 mg/kg	MSG + APV 50 mg/kg	MSG + APV 50 mg/kg	MSG + APV 50 mg/kg	MSG + APV 50 mg/kg
	F1	F2	F3	F4	F5	F6
P1	303	0	419	0	0	0
P2	0	0	0	0	0	0
P3	0	0	0	0	0	0
P4	0	0	0	0	0	0
P5	0	0	0	0	0	0
P6	155	0	0	0	0	249

	MSG + KYNA 10 mg/kg	MSG + KYNA 10 mg/kg	MSG + KYNA 10 mg/kg	MSG + KYNA 10 mg/kg	MSG + KYNA 10 mg/kg	MSG + KYNA 10 mg/kg
	F1	F2	F3	F4	F5	F6
P1	0	0	373	116	0	154
P2	0	0	0	19	0	0
P3	0	0	0	0	0	0
P4	0	0	0	0	0	0
P5	0	0	0	0	0	0
P6	40	0	0	0	0	0

	MSG + KYNA 50 mg/kg	MSG + KYNA 50 mg/kg	MSG + KYNA 50 mg/kg	MSG + KYNA 50 mg/kg	MSG + KYNA 50 mg/kg	MSG + KYNA 50 mg/kg
	F1	F2	F3	F4	F5	F6
P1	267	0	0	331	255	91
P2	131	0	0	0	0	0
P3	0	0	0	0	0	0
P4	167	113	0	0	70	0
P5	493	0	0	0	0	0
P6	505	0	0	0	75	0

	MSG + KYNA 100 mg/kg	MSG + KYNA 100 mg/kg	MSG + KYNA 100 mg/kg	MSG + KYNA 100 mg/kg	MSG + KYNA 100 mg/kg	MSG + KYNA 100 mg/kg
	F1	F2	F3	F4	F5	F6
P1	0	0	0	0	0	222
P2	0	0	0	16.5	0	0
P3	0	0	0	0	0	0
P4	0	0	0	0	0	0
P5	0	0	0	0	0	0
P6	0	0	0	0	0	0

	MSG + Olcegepant 10 mg/kg	SG + Olcegepant 10 mg/kg	SG + Olcegepant 10 mg/kg	SG + Olcegepant 10 mg/kg	SG + Olcegepant 10 mg/kg	SG + Olcegepant 10 mg/kg
	F1	F2	F3	F4	F5	F6
P1	0	0	0	0	0	0
P2	0	0	0	179	0	0
P3	0	0	0	177	0	0
P4	0	0	182	0	0	0
P5	0	0	0	0	0	0
P6	0	0	35	0	0	0

Lying-on-Belly (Male) - Study 1

	MSG + Vehicle	MSG + Vehicle	MSG + Vehicle	MSG + Vehicle	MSG + Vehicle	MSG + Vehicle
	M1	M2	M3	M4	M5	M6
P1	0	385	249	334	324	379
P2	0	475.5	0	0	0	271.5
P3	266.5	94	0	0	0	36.5
P4	0	0	0	0	0	0
P5	0	0	0	0	0	0
P6	0	0	0	0	0	0

	MSG + APV 50 mg/kg	MSG + APV 50 mg/kg	MSG + APV 50 mg/kg	MSG + APV 50 mg/kg	MSG + APV 50 mg/kg	MSG + APV 50 mg/kg
	M1	M2	M3	M4	M5	M6
P1	0	179	0	161	0	390
P2	0	0	0	0	0	617
P3	0	0	0	0	0	155
P4	0	0	0	0	0	0
P5	0	0	0	0	0	0
P6	0	0	0	0	0	0

	MSG + KYNA 10 mg/kg	MSG + KYNA 10 mg/kg	MSG + KYNA 10 mg/kg	MSG + KYNA 10 mg/kg	MSG + KYNA 10 mg/kg	MSG + KYNA 10 mg/kg
	M1	M2	M3	M4	M5	M6
P1	0	290	126	378	342	407
P2	0	310	322	503	369	573
P3	0	0	250	0	251	535
P4	0	20	0	0	0	0
P5	0	0	0	0	0	0
P6	0	500	0	0	0	0

	MSG + KYNA 50 mg/kg	MSG + KYNA 50 mg/kg	MSG + KYNA 50 mg/kg	MSG + KYNA 50 mg/kg	MSG + KYNA 50 mg/kg	MSG + KYNA 50 mg/kg
	M1	M2	M3	M4	M5	M6
P1	420	258	0	0	90	327
P2	0	562	0	0	363	592
P3	0	0	0	0	0	476
P4	0	0	0	0	0	28
P5	0	0	0	0	0	8
P6	0	0	0	0	0	0

	MSG + KYNA 100 mg/kg	MSG + KYNA 100 mg/kg	MSG + KYNA 100 mg/kg	MSG + KYNA 100 mg/kg	MSG + KYNA 100 mg/kg	MSG + KYNA 100 mg/kg
	M1	M2	M3	M4	M5	M6
P1	358.5	392.5	153.5	378	318.5	290
P2	0	572	0	502.5	290	361.5
P3	0	0	0	0	100.5	381
P4	0	0	0	0	0	0
P5	0	0	0	0	136	0
P6	0	0	0	0	0	0

	MSG + Olcegepant 10 mg/kg	SG + Olcegepant 10 mg/kg	SG + Olcegepant 10 mg/kg	SG + Olcegepant 10 mg/kg	SG + Olcegepant 10 mg/kg	SG + Olcegepant 10 mg/kg
	M1	M2	M3	M4	M5	M6
P1	0	0	0	0	0	125
P2	0	0	0	0	0	585
P3	0	0	0	415	124	206
P4	0	0	0	0	432	0
P5	0	0	0	0	0	0
P6	0	0	0	0	0	0

Mechanical Withdrawal Threshold - Study 1

	Time (min)	MSG + Vehicle	MSG + Vehicle	MSG + Vehicle	MSG + Vehicle	MSG + Vehicle	MSG + Vehicle	MSG + Vehicle	MSG + Vehicle	MSG + Vehicle	MSG + Vehicle	MSG + Vehicle	MSG + Vehicle
		F1	F2	F3	F4	F5	F6	M1	M2	M3	M4	M5	M6
B		1	1	1	1	1	1	1	1	1	1	1	1
P1	10	0.68	1.43	0.71	0.93	0.76	0.98	0.95	1.18	1.03	1.08	1	1.4
P2	20	0.5	1.65	1.17	1.18	0.92	1.18	1.65	1.14	1.5	1.17	1.14	1.51
P3	30	0.64	1.49	1.31	1.18	1.16	1.38	1.45	1.04	1.12	1.34	1.23	1.22
P4	40	3.24	1.6	1	0.67	1.04	1.12	1.25	1.17	1.39	1.26	0.97	1.48
P5	50	3.3	1.07	1.28	1.08	1.33	1.13	0.94	1.12	0.75	1	1.11	1.27
P6	60	0.68	1.13	1.01	1.3	0.85	1.2	1.17	1.19	1.21	1.48	0.87	1.31

	Time (min)	MSG + APV 50 mg/kg	MSG + APV 50 mg/kg	MSG + APV 50 mg/kg	MSG + APV 50 mg/kg	MSG + APV 50 mg/kg	MSG + APV 50 mg/kg	MSG + APV 50 mg/kg	MSG + APV 50 mg/kg	MSG + APV 50 mg/kg	MSG + APV 50 mg/kg	MSG + APV 50 mg/kg	MSG + APV 50 mg/kg
		F1	F2	F3	F4	F5	F6	M1	M2	M3	M4	M5	M6
B		1	1	1	1	1	1	1	1	1	1	1	1
P1	10	1.2	1.13	1.33	1.46	0.98	1.37	1.2	0.89	0.93	1.16	1.11	1.09
P2	20	1.7	1.23	1.27	0.99	0.63	1.03	1.79	0.96	0.99	1.17	0.75	1.22
P3	30	1.39	1.35	1.24	1.02	1.21	1.74	1.83	1.09	0.81	1.16	1.63	0.89
P4	40	1.49	1.12	1.22	1.28	0.95	1.44	1.7	0.99	0.75	1.12	1.52	1.15
P5	50	1.19	1.06	1.38	1.95	0.93	1.57	1.09	1	1.01	1.32	1.16	1.09
P6	60	1.66	1.01	1.31	1.22	0.88	1.3	1.76	1.2	1.01	1.63	0.79	1.14

	Time (min)	MSG + KYNA 10 mg/kg	MSG + KYNA 10 mg/kg	MSG + KYNA 10 mg/kg	MSG + KYNA 10 mg/kg	MSG + KYNA 10 mg/kg	MSG + KYNA 10 mg/kg	MSG + KYNA 10 mg/kg	MSG + KYNA 10 mg/kg	MSG + KYNA 10 mg/kg	MSG + KYNA 10 mg/kg	MSG + KYNA 10 mg/kg	MSG + KYNA 10 mg/kg
		F1	F2	F3	F4	F5	F6	M1	M2	M3	M4	M5	M6
B		1	1	1	1	1	1	1	1	1	1	1	1
P1	10	0.78	1.62	1.51	0.66	0.7	1.32	1.37	0.72	1.82	0.86	1.08	1.39
P2	20	0.77	1.66	1.39	0.85	0.89	1.36	1.95	0.68	1.89	1.23	1.13	1.15
P3	30	0.62	1.4	1.99	0.86	0.87	1.3	2	0.7	2.15	1.08	0.66	1.19
P4	40	0.55	1.4	2.12	0.64	0.9	0.81	1.18	0.78	2.1	0.93	0.87	1
P5	50	0.68	1.13	1.81	1.02	1.12	0.75	1.37	0.68	2.13	0.96	1.1	0.92
P6	60	0.59	1.17	1.9	0.98	1.02	0.84	1.26	0.68	2.19	1.06	0.97	1.33

	Time (min)	MSG + KYNA 50 mg/kg	MSG + KYNA 50 mg/kg	MSG + KYNA 50 mg/kg	MSG + KYNA 50 mg/kg	MSG + KYNA 50 mg/kg	MSG + KYNA 50 mg/kg	MSG + KYNA 50 mg/kg	MSG + KYNA 50 mg/kg	MSG + KYNA 50 mg/kg	MSG + KYNA 50 mg/kg	MSG + KYNA 50 mg/kg	MSG + KYNA 50 mg/kg
		F1	F2	F3	F4	F5	F6	M1	M2	M3	M4	M5	M6
B		1	1	1	1	1	1	1	1	1	1	1	1
P1	10	1.43	1.3	1.49	0.96	1.31	0.9	0.91	0.98	1.37	1.2	0.91	1.43
P2	20	1.36	1.25	1.16	0.96	0.74	1.1	0.9	1.63	1.13	1.29	0.86	1.45
P3	30	0.94	1.21	1.12	0.71	0.86	1.88	1.44	1.19	1.4	0.76	0.75	1.33
P4	40	1.24	1.32	1.38	0.82	1.22	1.65	1.38	1.08	1.21	0.75	0.97	1.4
P5	50	1.12	1.26	1.23	1.36	1.09	1.26	1.38	1.16	1.4	1.03	1	1.15
P6	60	0.91	1.31	0.95	1.24	1.1	0.95	1.45	1.26	1.54	1.3	0.81	1.3

	Time (min)	MSG + KYNA 100 mg/kg	MSG + KYNA 100 mg/kg	MSG + KYNA 100 mg/kg	MSG + KYNA 100 mg/kg	MSG + KYNA 100 mg/kg	MSG + KYNA 100 mg/kg	MSG + KYNA 100 mg/kg	MSG + KYNA 100 mg/kg	MSG + KYNA 100 mg/kg	MSG + KYNA 100 mg/kg	MSG + KYNA 100 mg/kg	MSG + KYNA 100 mg/kg
		F1	F2	F3	F4	F5	F6	M1	M2	M3	M4	M5	M6
B		1	1	1	1	1	1	1	1	1	1	1	1
P1	10	1.05	2	1.48	1.24	1.66	0.99	0.69	1.04	1.46	0.68	0.81	1.04
P2	20	1.04	1.87	1.16	1.13	1.43	1.07	0.81	1.2	1.5	0.79	0.84	0.71
P3	30	1.1	1.83	1.92	1.1	0.89	1.13	0.65	1.14	1.21	0.77	1.02	0.85
P4	40	0.95	1.43	1.52	0.93	1.31	1.11	1.11	0.78	1.4	0.62	0.69	0.92
P5	50	1.02	1.22	1.35	1.1	1.23	0.93	0.85	1.4	1.19	0.98	0.98	0.69
P6	60	1.12	0.81	1.25	1.05	1.04	0.95	1.02	1.4	1.21	0.74	0.94	0.98

	Time (min)	MSG + Olcegepant 1 mg/kg	MSG + Olcegepant 1 mg/kg	MSG + Olcegepant 1 mg/kg	MSG + Olcegepant 1 mg/kg	MSG + Olcegepant 1 mg/kg	MSG + Olcegepant 1 mg/kg	MSG + Olcegepant 1 mg/kg	MSG + Olcegepant 1 mg/kg	MSG + Olcegepant 1 mg/kg	MSG + Olcegepant 1 mg/kg	MSG + Olcegepant 1 mg/kg	MSG + Olcegepant 1 mg/kg
		F1	F2	F3	F4	F5	F6	M1	M2	M3	M4	M5	M6
B		1	1	1	1	1	1	1	1	1	1	1	1
P1	10	1.29	1.37	0.91	1.04	0.92	1.39	1.13	1.27	1.63	1.14	0.83	1.01
P2	20	1.15	1.79	0.81	0.83	1.31	1.57	1.36	0.68	2.04	1.27	0.9	1.2
P3	30	1.29	1.56	0.83	1.14	0.82	0.95	0.86	0.69	2.18	1.52	1.28	1.17
P4	40	1.21	1.2	0.95	0.89	1.56	1.63	1.15	0.92	1.91	1.43	0.69	1.26
P5	50	1.66	0.77	1.12	0.73	1.73	1.13	0.87	0.62	2	1.05	0.8	1.34
P6	60	1.08	1.19	1.06	0.78	0.97	1.44	1.26	0.92	2.01	1.42	0.98	1.37

Rearing - Study 1

	Time (min)	MSG + Vehicle	MSG + Vehicle	MSG + Vehicle	MSG + Vehicle	MSG + Vehicle	MSG + Vehicle	MSG + Vehicle	MSG + Vehicle	MSG + Vehicle	MSG + Vehicle	MSG + Vehicle	MSG + Vehicle
		F1	F2	F3	F4	F5	F6	M1	M2	M3	M4	M5	M6
P1	10	0	0	0	0	0	0.252	0.392	0	0	0.257	0.01	0
P2	20	0.074	0.011	0	0	0	0.325	0.411	0	0	0.414	0.103	0
P3	30	0.874	0	0	0.073	0.362	0.477	0.354	0	0	0.757	0.123	0
P4	40	0	0	0	0.411	0.082	0.271	0.417	0	0	1.873	0.071	0.061
P5	50	0	0.028	0	0.28	0.42	0.398	0.417	0	0	0.199	0.706	0.055
P6	60	0	0	0	0.976	0.754	0.836	0.348	0.019	0	2.303	0.225	0.009

	Time (min)	MSG + APV 50 mg/kg	MSG + APV 50 mg/kg	MSG + APV 50 mg/kg	MSG + APV 50 mg/kg	MSG + APV 50 mg/kg	MSG + APV 50 mg/kg	MSG + APV 50 mg/kg	MSG + APV 50 mg/kg	MSG + APV 50 mg/kg	MSG + APV 50 mg/kg	MSG + APV 50 mg/kg	MSG + APV 50 mg/kg
		F1	F2	F3	F4	F5	F6	M1	M2	M3	M4	M5	M6
P1	10	0.24	0.302	0.35	0.63	1.889	1.239	0.216	0.23	0.176	0.14	0.75	0
P2	20	0	0.15	0	0.472	0.694	0.614	0.272	0	0.035	0.149	0.014	0
P3	30	0.085	0.687	0	0.244	0.369	1.39	0.208	0.057	0.365	0.624	0.085	0
P4	40	0	0.008	0	0.259	0	3.054	0.352	0.141	0.025	1.115	0.091	0.031
P5	50	0.037	0.178	0	0.377	0.228	2.96	0.712	0	0.032	0.236	0.289	0.066
P6	60	0	0.061	0	0.392	0.334	1.034	0.104	0.255	0.014	0.22	0.536	0.012

	Time (min)	MSG + KYNA 10 mg/kg	MSG + KYNA 10 mg/kg	MSG + KYNA 10 mg/kg	MSG + KYNA 10 mg/kg	MSG + KYNA 10 mg/kg	MSG + KYNA 10 mg/kg	MSG + KYNA 10 mg/kg	MSG + KYNA 10 mg/kg	MSG + KYNA 10 mg/kg	MSG + KYNA 10 mg/kg	MSG + KYNA 10 mg/kg	MSG + KYNA 10 mg/kg
		F1	F2	F3	F4	F5	F6	M1	M2	M3	M4	M5	M6
P1	10	1.205	0	0	0.059	1.913	0.89	0.89	0.01	0	0	0.11	0
P2	20	0.087	0.106	0	0.155	0.667	2.384	2.384	0	0	0.008	0.147	0
P3	30	0.268	0.641	0	0	1.232	0.265	0.265	0.063	0.017	0.072	0.125	0
P4	40	0.551	1.24	0.075	0.262	2.091	0.07	0.07	0.03	0	0.325	0.151	0
P5	50	0.236	1.013	0.248	0.15	0.251	0.078	0.078	0.361	0	0.122	0.629	0
P6	60	0	1.002	0	0	0.354	0	0	0	0	0.038	1.621	0

	Time (min)	MSG + KYNA 50 mg/kg	MSG + KYNA 50 mg/kg	MSG + KYNA 50 mg/kg	MSG + KYNA 50 mg/kg	MSG + KYNA 50 mg/kg	MSG + KYNA 50 mg/kg	MSG + KYNA 50 mg/kg	MSG + KYNA 50 mg/kg	MSG + KYNA 50 mg/kg	MSG + KYNA 50 mg/kg	MSG + KYNA 50 mg/kg	MSG + KYNA 50 mg/kg
		F1	F2	F3	F4	F5	F6	M1	M2	M3	M4	M5	M6
P1	10	0.934	0	0.251	0.157	0	0	0.12	0	0	0.65	0.039	0.01
P2	20	0	0	1.214	0	0.013	0.118	0.408	0	0	0.967	0.027	0
P3	30	0	0	1.236	0.176	0.035	0.097	0.045	0.047	0	0.284	0.067	0
P4	40	0	0.075	0.382	0	0	0.14	0.061	0	0	0.128	0.092	0
P5	50	0	0.014	0	0.32	0.013	0.053	0.03	0.154	0	0.728	0.153	0
P6	60	0	0.003	0	0.111	0.125	0.388	0.669	0	0	0	0.388	0.031

	Time (min)	MSG + KYNA 100 mg/kg	MSG + KYNA 100 mg/kg	MSG + KYNA 100 mg/kg	MSG + KYNA 100 mg/kg	MSG + KYNA 100 mg/kg	MSG + KYNA 100 mg/kg	MSG + KYNA 100 mg/kg	MSG + KYNA 100 mg/kg	MSG + KYNA 100 mg/kg	MSG + KYNA 100 mg/kg	MSG + KYNA 100 mg/kg	MSG + KYNA 100 mg/kg
		F1	F2	F3	F4	F5	F6	M1	M2	M3	M4	M5	M6
P1	10	0	0	0.101	0	0	0	0.006	0	0	0.039	0.016	0
P2	20	0	0	0.389	0.067	0.079	0.019	0	0	0	0	0.03	0
P3	30	0.876	0	0.732	0	0	0.047	0	0	0	0	0.028	0
P4	40	0.473	2.146	0.176	0.029	0.087	0	0.029	0.053	0	0.092	0.179	0
P5	50	0.039	1.092	0.074	0.114	0.154	0.036	0.42	0.063	0.108	0	0.089	0.013
P6	60	0.085	4.552	2.881	0	0.288	0	0.236	0	0.264	0.026	0.086	0.023

	Time (min)	Olcegepant 10 mg/kg	Olcegepant 10 mg/kg	Olcegepant 10 mg/kg	Olcegepant 10 mg/kg	Olcegepant 10 mg/kg	Olcegepant 10 mg/kg	Olcegepant 10 mg/kg	Olcegepant 10 mg/kg	Olcegepant 10 mg/kg	Olcegepant 10 mg/kg	Olcegepant 10 mg/kg	Olcegepant 10 mg/kg
		F1	F2	F3	F4	F5	F6	M1	M2	M3	M4	M5	M6
P1	10	0.191	0	0.015	0.007	0	0	0.018	0.019	0	0.042	0	0.029
P2	20	0	0	2.577	0.01	0.236	0.184	0	0	0.109	0.059	0.057	0
P3	30	0.117	0	0.546	0.028	0.274	0.32	0.099	0.213	0	0.084	0.084	0.022
P4	40	0.371	0.045	0.734	0	0.359	1.137	0.181	0.007	0	0.064	0.364	0.073
P5	50	0.107	0	0.384	0	0	0.466	0	0	0.113	0	0.163	0.036
P6	60	0.032	0	0.133	0	0.182	0.242	0	0.29	0.055	0.947	1.147	0.029

Rotarod (RPM) - Study 2

	Time (min)	Saline	Saline	Saline	Saline	Saline	Saline
		F1	F2	F3	M1	M2	M3
P1	5	1.61	1.22	0.78	0.89	1.21	0.76
P2	20	1.15	1.28	0.74	1.07	1.21	1.35
P3	35	0.93	0.86	0.73	0.77	1.29	1.3

	Time (min)	KYNA 100 mg/kg	KYNA 100 mg/kg	KYNA 100 mg/kg	KYNA 100 mg/kg	KYNA 100 mg/kg	KYNA 100 mg/kg
		F1	F2	F3	M1	M2	M3
P1	5	1.09	0.83	0.88	1.34	0.84	1.21
P2	20	0.65	1.53	0.91	1.55	0.88	1.75
P3	35	0.77	1.13	1.02	1.39	0.68	1.31

	Time (min)	APV 50 mg/kg	APV 50 mg/kg	APV 50 mg/kg	APV 50 mg/kg	APV 50 mg/kg	APV 50 mg/kg
		F1	F2	F3	M1	M2	M3
P1	5	1.42	0.97	0.76	0.97	1.3	0.59
P2	20	1.03	0.98	1.06	1.06	1.43	0.88
P3	35	1.97	1.24	1.17	1.18	1.66	0.93

	Time (min)	Olcegepant 1 mg/kg	Olcegepant 1 mg/kg	Olcegepant 1 mg/kg	Olcegepant 1 mg/kg	Olcegepant 1 mg/kg	Olcegepant 1 mg/kg
		F1	F2	F3	M1	M2	M3
P1	5	0.34	1.05	1.2	1.53	0.77	1.36
P2	20	0.28	1.06	0.91	1.13	0.94	1.3
P3	35	0.32	0.95	1.28	1.13	1	1.18

Rotarod (Latency) - Study 2

	Time (min)	Saline	Saline	Saline	Saline	Saline	Saline
		F1	F2	F3	M1	M2	M3
P1	5	2.82	1.27	1.34	0.85	1.29	0.62
P2	20	1.44	1.33	1.24	1.08	1.29	1.53
P3	35	0.78	0.83	1.22	0.68	1.42	1.47

	Time (min)	KYNA 100 mg/kg	KYNA 100 mg/kg	KYNA 100 mg/kg	KYNA 100 mg/kg	KYNA 100 mg/kg	KYNA 100 mg/kg
		F1	F2	F3	M1	M2	M3
P1	5	1.12	0.8	0.86	1.49	0.78	1.32
P2	20	0.51	1.65	0.89	1.81	0.84	2.18
P3	35	0.69	1.68	1.02	1.58	0.58	1.49

	Time (min)	APV 50 mg/kg	APV 50 mg/kg	APV 50 mg/kg	APV 50 mg/kg	APV 50 mg/kg	APV 50 mg/kg
		F1	F2	F3	M1	M2	M3
P1	5	1.99	0.96	0.7	0.96	1.46	0.42
P2	20	1.05	0.98	1.08	1.08	1.67	0.83
P3	35	3.28	1.29	1.21	1.24	2.02	0.9

	Time (min)	Olcegepant 1 mg/kg	Olcegepant 1 mg/kg	Olcegepant 1 mg/kg	Olcegepant 1 mg/kg	Olcegepant 1 mg/kg	Olcegepant 1 mg/kg
		F1	F2	F3	M1	M2	M3
P1	5	0.19	1.05	1.27	1.71	0.71	1.53
P2	20	0.1	1.07	0.87	1.97	0.93	1.44
P3	35	0.15	0.95	1.38	2.05	0.99	1.24

Rotarod (Distance Travelled) - Study 2

	Time (min)	Saline	Saline	Saline	Saline	Saline	Saline
		F1	F2	F3	M1	M2	M3
P1	5	3.87	1.5	0.55	0.76	1.47	0.49
P2	20	1.57	1.63	0.48	1.12	1.48	1.85
P3	35	0.77	0.72	0.47	0.54	1.71	1.73

	Time (min)	KYNA 100 mg/kg	KYNA 100 mg/kg	KYNA 100 mg/kg	KYNA 100 mg/kg	KYNA 100 mg/kg	KYNA 100 mg/kg
		F1	F2	F3	M1	M2	M3
P1	5	1.13	0.6	0.73	1.85	0.64	1.53
P2	20	0.35	2.11	0.79	2.51	0.72	3.36
P3	35	0.53	2.18	0.98	2.01	0.41	1.83

	Time (min)	APV 50 mg/kg	APV 50 mg/kg	APV 50 mg/kg	APV 50 mg/kg	APV 50 mg/kg	APV 50 mg/kg
		F1	F2	F3	M1	M2	M3
P1	5	2.43	0.93	0.54	0.93	1.74	0.28
P2	20	1.05	0.96	1.09	1.12	2.12	0.75
P3	35	5.05	1.54	1.33	1.41	2.91	0.84

	Time (min)	Olcegepant 1 mg/kg	Olcegepant 1 mg/kg	Olcegepant 1 mg/kg	Olcegepant 1 mg/kg	Olcegepant 1 mg/kg	Olcegepant 1 mg/kg
		F1	F2	F3	M1	M2	M3
P1	5	0.07	1.09	1.37	2.42	0.55	1.85
P2	20	0.04	1.13	0.77	2.39	0.86	1.67
P3	35	0.06	0.9	1.57	1.29	0.96	1.34

Rearing - Study 3

	Time (min)	MSG + Vehicle M1	MSG + Vehicle M2	MSG + Vehicle M3	MSG + Vehicle M4	MSG + Vehicle M5	MSG + Vehicle M6
P1	10	0	0.33	0	1.13	0	0.03
P2	20	0.05	0.02	0	0.29	0.15	0
P3	30	0	0.06	0.06	0.18	0.05	0
P4	40	0	0.12	0.03	0.47	0.02	0
P5	50	0	0.69	0.02	0.02	0.03	0.05
P6	60	0.19	0.93	0	0	0	0

	Time (min)	MSG + MTC 3 mg/kg M1	MSG + MTC 3 mg/kg M2	MSG + MTC 3 mg/kg M3	MSG + MTC 3 mg/kg M4	MSG + MTC 3 mg/kg M5	MSG + MTC 3 mg/kg M6
P1	10	0.008	0.181	0.064	0	0.663	0
P2	20	0.132	0.039	0.109	0.126	0.392	0.081
P3	30	0.199	0.105	0.275	0.184	0	0.147
P4	40	0.311	0.031	0.543	0.146	0.038	0
P5	50	0.253	0.102	0.632	2.711	0	0
P6	60	0.463	0.111	1.625	5.041	0.058	0

	Time (min)	MSG + OND 0.5 mg/kg M1	MSG + OND 0.5 mg/kg M2	MSG + OND 0.5 mg/kg M3	MSG + OND 0.5 mg/kg M4	MSG + OND 0.5 mg/kg M5	MSG + OND 0.5 mg/kg M6
P1	10	0.008	0.184	0.031	0	0.903	0
P2	20	0.132	0.301	0	0	0	0
P3	30	0.199	0.672	0.038	0.013	0	2.136
P4	40	0.311	0.808	0.158	0.154	0.058	0
P5	50	0.253	1.293	0.045	0.071	0.185	0
P6	60	0.463	0.934	0.095	0.411	1.643	0

Mechanical Withdrawal Threshold - Study 3

	Time (min)	MSG + Vehicle	MSG + Vehicle	MSG + Vehicle	MSG + Vehicle	MSG + Vehicle	MSG + Vehicle
		M1	M2	M3	M4	M5	M6
P1	10	1.08	1.51	1.12	0.83	1.34	0.7
P2	20	1.06	1.29	1.12	0.75	1.41	0.75
P3	30	1.1	1.42	1.24	0.7	1.52	0.7
P4	40	0.86	0.86	1.31	0.84	1.5	0.84
P5	50	1.14	0.93	1.17	1.01	1.31	0.71
P6	60	0.98	0.8	1.3	0.7	1.37	0.4

	Time (min)	MSG + MTC 3 mg/kg	MSG + MTC 3 mg/kg	MSG + MTC 3 mg/kg	MSG + MTC 3 mg/kg	MSG + MTC 3 mg/kg	MSG + MTC 3 mg/kg
		M1	M2	M3	M4	M5	M6
P1	10	1.31	1.02	0.92	0.9	1.19	1.48
P2	20	1.15	1.32	0.95	1.43	1.13	1.44
P3	30	1.01	0.91	1.1	1.21	1.16	1.58
P4	40	1.22	1.01	1.44	1.26	1.22	1.01
P5	50	1.06	0.86	0.88	1.43	1.44	1.41
P6	60	0.88	0.77	0.9	1.71	1.34	1.28

	Time (min)	MSG + OND 0.5 mg/kg	MSG + OND 0.5 mg/kg	MSG + OND 0.5 mg/kg	MSG + OND 0.5 mg/kg	MSG + OND 0.5 mg/kg	MSG + OND 0.5 mg/kg
		M1	M2	M3	M4	M5	M6
P1	10	0.96	0.67	1.05	1.43	1.77	1.25
P2	20	1.14	0.6	1.01	1.27	1.16	1.16
P3	30	1.31	1.07	1.04	1.36	1.43	1.16
P4	40	1.13	1.3	0.9	1.48	1.82	1.16
P5	50	1.15	1.04	0.98	1.68	1.2	1.17
P6	60	1.06	1.18	0.98	1.64	1.75	1.2

Facial Grooming - Study 3

	Time (min)	MSG + Vehicle M1	MSG + Vehicle M2	MSG + Vehicle M3	MSG + Vehicle M4	MSG + Vehicle M5	MSG + Vehicle M6
P1	10	0	1.577	0	1.069	0	0
P2	20	0.108	0	0.559	0.732	0.432	0
P3	30	0.123	0	0.162	0	0.915	0.233
P4	40	0.088	0.867	1.235	0.071	1.276	0.455
P5	50	0	3.128	0	0	0.26	0
P6	60	0.549	1.645	0	0.183	0	0

	Time (min)	MSG + MTC 3 mg/kg M1	MSG + MTC 3 mg/kg M2	MSG + MTC 3 mg/kg M3	MSG + MTC 3 mg/kg M4	MSG + MTC 3 mg/kg M5	MSG + MTC 3 mg/kg M6
P1	10	0	3.317	0.58	0	6.736	0.1
P2	20	0.563	0	0	0.25	5.764	0
P3	30	0.757	0	0.729	0	6.364	0.4
P4	40	0.25	0.25	2.217	0.556	0	0
P5	50	0	0	1.091	0.76	0	1.908
P6	60	0.688	0.833	0.73	0.486	0	0

	Time (min)	MSG + OND 0.5 mg/kg M1	MSG + OND 0.5 mg/kg M2	MSG + OND 0.5 mg/kg M3	MSG + OND 0.5 mg/kg M4	MSG + OND 0.5 mg/kg M5	MSG + OND 0.5 mg/kg M6
P1	10	0.056	3.028	0.254	0	1.625	0
P2	20	0.197	0	0.287	0.032	0	0
P3	30	0.693	1.586	0.076	0.613	0	1.892
P4	40	0	0.668	1.164	0	0.891	0
P5	50	1.349	0.767	0.526	0.387	0	0
P6	60	0.211	0	0.607	0	1.058	0

Rat Grimace Score - Study 3

	Time (min)	MSG + Vehicle	MSG + Vehicle	MSG + Vehicle	MSG + Vehicle	MSG + Vehicle	MSG + Vehicle
		M1	M2	M3	M4	M5	M6
P1	10	2	2	2	0.25	1.25	0.5
P2	20	1.5	2	2	0.5	1.5	0.25
P3	30	0.5	1.5	1.75	0	0	0
P4	40	0.5	0	1	0	0	0.25
P5	50	0	0	0	0	0	0.75
P6	60	0	0	0	0	0	0.75

	Time (min)	MSG + MTC 3 mg/kg	MSG + MTC 3 mg/kg	MSG + MTC 3 mg/kg	MSG + MTC 3 mg/kg	MSG + MTC 3 mg/kg	MSG + MTC 3 mg/kg
		M1	M2	M3	M4	M5	M6
P1	10	0	1	1.75	1.5	0.25	0.75
P2	20	0	1	1.5	0	0.25	1.25
P3	30	0	1	0	0	0	0.25
P4	40	0.25	0.25	0	0	0	1
P5	50	0	0	0	0	0	0.75
P6	60	0	0	0	0	0	0

	Time (min)	MSG + OND 0.5 mg/kg	MSG + OND 0.5 mg/kg	MSG + OND 0.5 mg/kg	MSG + OND 0.5 mg/kg	MSG + OND 0.5 mg/kg	MSG + OND 0.5 mg/kg
		M1	M2	M3	M4	M5	M6
P1	10	1	0	2	2	1	1.75
P2	20	0	0	1.75	1	0.25	1.75
P3	30	0.25	0	2	0.75	0	0.75
P4	40	0	0.25	1	0.75	0	0.75
P5	50	0.25	0.25	0.5	1.5	0	0
P6	60	0	0	0.25	0	0	0

Head Flicks - Study 3

	Time (min)	MSG + Vehicle	MSG + Vehicle	MSG + Vehicle	MSG + Vehicle	MSG + Vehicle	MSG + Vehicle
		M1	M2	M3	M4	M5	M6
P1	10	16	4	14	0	16	14
P2	20	6	3	5	0	5	5
P3	30	1	2	2	0	2	0
P4	40	3	0	0	0	0	0
P5	50	0	0	0	0	0	0
P6	60	0	0	0	0	0	0

	Time (min)	MSG + MTC 3 mg/kg	MSG + MTC 3 mg/kg	MSG + MTC 3 mg/kg	MSG + MTC 3 mg/kg	MSG + MTC 3 mg/kg	MSG + MTC 3 mg/kg
		M1	M2	M3	M4	M5	M6
P1	10	8	12	2	9	0	9
P2	20	0	5	0	1	0	2
P3	30	0	2	0	0	0	0
P4	40	3	0	0	1	0	0
P5	50	0	0	0	0	0	0
P6	60	0	0	0	0	0	0

	Time (min)	MSG + OND 0.5 mg/kg	MSG + OND 0.5 mg/kg	MSG + OND 0.5 mg/kg	MSG + OND 0.5 mg/kg	MSG + OND 0.5 mg/kg	MSG + OND 0.5 mg/kg
		M1	M2	M3	M4	M5	M6
P1	10	6	0	18	13	2	6
P2	20	0	0	0	2	0	0
P3	30	0	0	0	3	0	0
P4	40	0	0	0	2	0	0
P5	50	0	0	0	0	0	0
P6	60	0	0	0	0	0	0

Head Scratches - Study 3

	Time (min)	MSG + Vehicle	MSG + Vehicle	MSG + Vehicle	MSG + Vehicle	MSG + Vehicle	MSG + Vehicle
		M1	M2	M3	M4	M5	M6
P1	10	0	0.25	0	0.893	0	0
P2	20	0	0	0.25	0	0.277	0
P3	30	0	0	0	0	0.186	0.05
P4	40	0	0.292	0.417	0	0.291	0.05
P5	50	0	1.75	0	0	0.091	0
P6	60	0.385	1.167	0	0	0	0

	Time (min)	MSG + MTC 3 mg/kg	MSG + MTC 3 mg/kg	MSG + MTC 3 mg/kg	MSG + MTC 3 mg/kg	MSG + MTC 3 mg/kg	MSG + MTC 3 mg/kg
		M1	M2	M3	M4	M5	M6
P1	10	0	1.75	0.372	0	1.909	0.113
P2	20	0	0	0.278	0.136	0.909	0
P3	30	0.958	0	0.539	0	1.682	0.238
P4	40	0.167	0	1.622	0.091	0	0
P5	50	0	0	0.889	0.36	0	1.288
P6	60	1.333	1	0.322	0.279	0	0

	Time (min)	MSG + OND 0.5 mg/kg	MSG + OND 0.5 mg/kg	MSG + OND 0.5 mg/kg	MSG + OND 0.5 mg/kg	MSG + OND 0.5 mg/kg	MSG + OND 0.5 mg/kg
		M1	M2	M3	M4	M5	M6
P1	10	0.05	1.367	0.063	0	1.837	0.125
P2	20	0.1	0	0.292	0	0	0
P3	30	0.183	0.3	0	0.404	0	0.688
P4	40	0	0.367	1.521	0	0.397	0
P5	50	0.517	0.133	0.701	0.333	0	0
P6	60	0.067	0	0.778	0	0.845	0

Lying-on-Belly - Study 3

	Time (min)	MSG + Vehicle	MSG + Vehicle	MSG + Vehicle	MSG + Vehicle	MSG + Vehicle	MSG + Vehicle
		M1	M2	M3	M4	M5	M6
P1	10	393.5	117.5	447.5	0	404.5	310.5
P2	20	278.5	189	334.5	44	120	556
P3	30	545	25	174.5	0	231	395
P4	40	265.5	63	339	0	55.5	150
P5	50	526.5	15.5	490	3.5	297	168.5
P6	60	0	46	404	0	363	87.5

	Time (min)	MSG + MTC 3 mg/kg	MSG + MTC 3 mg/kg	MSG + MTC 3 mg/kg	MSG + MTC 3 mg/kg	MSG + MTC 3 mg/kg	MSG + MTC 3 mg/kg
		M1	M2	M3	M4	M5	M6
P1	10	284.5	0	175.5	103.5	0	286
P2	20	37	0	97.5	126	0	150
P3	30	223.5	0	34.5	3	12.5	477.5
P4	40	18.5	0	5	0	242	90
P5	50	0	0	115	0	0	36.5
P6	60	0	0	51.5	0	152	249

	Time (min)	MSG + OND 0.5 mg/kg	MSG + OND 0.5 mg/kg	MSG + OND 0.5 mg/kg	MSG + OND 0.5 mg/kg	MSG + OND 0.5 mg/kg	MSG + OND 0.5 mg/kg
		M1	M2	M3	M4	M5	M6
P1	10	373	0	284	313.5	4.5	406
P2	20	0	0	332.5	150	0	510
P3	30	0	0	172.5	60	0	0
P4	40	0	0	15.5	499.5	0	314
P5	50	0	0	150	44.5	90	342.5
P6	60	0	0	19	233.5	22	240

Sex Difference – Grimace Score

Type of Statistical Test	Statistical Details	Multiple Comparisons	P Value
Two-way RM ANOVA with Tukey's multiple comparison test	Time factor: F(3.486, 209.2) = 41.45; Sex factor: F(11, 60) = 1.789; Time x Treatment Factor: F(55, 300) = 0.9891	**P1**	
		MSG + Vehicle (Female) vs. MSG + Vehicle (Male)	0.929
		MSG + APV 50 mg/kg (Female) vs. MSG + APV 50 mg/kg (Male)	0.998
		MSG + KYNAA 10 mg/kg (Female) vs. MSG + KYNAA 10 mg/kg (Male)	0.980
		MSG + KYNAA 50 mg/kg (Female) vs. MSG + KYNAA 50 mg/kg (Male)	>0.9999
		MSG + KYNAA 100 mg/kg (Female) vs. MSG + KYNAA 100 mg/kg (Male)	>0.9999
		**P2**	
		MSG + Vehicle (Female) vs. MSG + Vehicle (Male)	0.682
		MSG + APV 50 mg/kg (Female) vs. MSG + APV 50 mg/kg (Male)	>0.9999
		MSG + KYNAA 10 mg/kg (Female) vs. MSG + KYNAA 10 mg/kg (Male)	0.998
		MSG + KYNAA 50 mg/kg (Female) vs. MSG + KYNAA 50 mg/kg (Male)	0.996
		MSG + KYNAA 100 mg/kg (Female) vs. MSG + KYNAA 100 mg/kg (Male)	0.997
		**P3**	
		MSG + Vehicle (Female) vs. MSG + Vehicle (Male)	0.944
		MSG + APV 50 mg/kg (Female) vs. MSG + APV 50 mg/kg (Male)	0.995
		MSG + KYNAA 10 mg/kg (Female) vs. MSG + KYNAA 10 mg/kg (Male)	0.974
		MSG + KYNAA 50 mg/kg (Female) vs. MSG + KYNAA 50 mg/kg (Male)	0.999
		MSG + KYNAA 100 mg/kg (Female) vs. MSG + KYNAA 100 mg/kg (Male)	>0.9999
		**P4**	
		MSG + Vehicle (Female) vs. MSG + Vehicle (Male)	0.941
		MSG + APV 50 mg/kg (Female) vs. MSG + APV 50 mg/kg (Male)	0.933
		MSG + KYNAA 10 mg/kg (Female) vs. MSG + KYNAA 10 mg/kg (Male)	0.818
		MSG + KYNAA 50 mg/kg (Female) vs. MSG + KYNAA 50 mg/kg (Male)	>0.9999
		MSG + KYNAA 100 mg/kg (Female) vs. MSG + KYNAA 100 mg/kg (Male)	0.986
		**P5**	
		MSG + Vehicle (Female) vs. MSG + Vehicle (Male)	0.784
		MSG + APV 50 mg/kg (Female) vs. MSG + APV 50 mg/kg (Male)	0.892
		MSG + KYNAA 10 mg/kg (Female) vs. MSG + KYNAA 10 mg/kg (Male)	0.771
		MSG + KYNAA 50 mg/kg (Female) vs. MSG + KYNAA 50 mg/kg (Male)	>0.9999
		MSG + KYNAA 100 mg/kg (Female) vs. MSG + KYNAA 100 mg/kg (Male)	0.929
		**P6**	
		MSG + Vehicle (Female) vs. MSG + Vehicle (Male)	0.588
		MSG + APV 50 mg/kg (Female) vs. MSG + APV 50 mg/kg (Male)	0.824
		MSG + KYNAAA 10 mg/kg (Female) vs. MSG + KYNAA 10 mg/kg (Male)	0.747
		MSG + KYNAA 50 mg/kg (Female) vs. MSG + KYNAA 50 mg/kg (Male)	0.989
		MSG + KYNAA 100 mg/kg (Female) vs. MSG + KYNAA 100 mg/kg (Male)	>0.9999

Sex Difference – Head Flick

Type of Statistical Test	Statistical Details	Multiple Comparisons	P Value
Two-way RM ANOVA with Tukey's multiple comparison test	Time factor: $F(2.173, 130.4) = 36.91$; Sex factor: $F(11, 60) = 2.221$; Time x Treatment Factor: $F(55, 300) = 1.955$	P1	
		MSG + Vehicle (Female) vs. MSG + Vehicle (Male)	>0.9999
		MSG + APV 50 mg/kg (Female) vs. MSG + APV 50 mg/kg (Male)	>0.9999
		MSG + KYNA 10 mg/kg (Female) vs. MSG + KYNA 10 mg/kg (Male)	>0.9999
		MSG + KYNA 50 mg/kg (Female) vs. MSG + KYNA 50 mg/kg (Male)	>0.9999
		MSG + KYNA 100 mg/kg (Female) vs. MSG + KYNA 100 mg/kg (Male)	0.927
		P2	
		MSG + Vehicle (Female) vs. MSG + Vehicle (Male)	>0.9999
		MSG + APV 50 mg/kg (Female) vs. MSG + APV 50 mg/kg (Male)	>0.9999
		MSG + KYNA 10 mg/kg (Female) vs. MSG + KYNA 10 mg/kg (Male)	>0.9999
		MSG + KYNA 50 mg/kg (Female) vs. MSG + KYNA 50 mg/kg (Male)	0.979
		MSG + KYNA 100 mg/kg (Female) vs. MSG + KYNA 100 mg/kg (Male)	>0.9999
		P3	
		MSG + Vehicle (Female) vs. MSG + Vehicle (Male)	>0.9999
		MSG + APV 50 mg/kg (Female) vs. MSG + APV 50 mg/kg (Male)	>0.9999
		MSG + KYNA 10 mg/kg (Female) vs. MSG + KYNA 10 mg/kg (Male)	>0.9999
		MSG + KYNA 50 mg/kg (Female) vs. MSG + KYNA 50 mg/kg (Male)	>0.9999
		MSG + KYNA 100 mg/kg (Female) vs. MSG + KYNA 100 mg/kg (Male)	>0.9999
		P4	
		MSG + Vehicle (Female) vs. MSG + Vehicle (Male)	>0.9999
		MSG + APV 50 mg/kg (Female) vs. MSG + APV 50 mg/kg (Male)	>0.9999
		MSG + KYNA 10 mg/kg (Female) vs. MSG + KYNA 10 mg/kg (Male)	>0.9999
		MSG + KYNA 50 mg/kg (Female) vs. MSG + KYNA 50 mg/kg (Male)	0.971
		MSG + KYNA 100 mg/kg (Female) vs. MSG + KYNA 100 mg/kg (Male)	>0.9999
		P5	
		MSG + Vehicle (Female) vs. MSG + Vehicle (Male)	>0.9999
		MSG + APV 50 mg/kg (Female) vs. MSG + APV 50 mg/kg (Male)	>0.9999
		MSG + KYNA 10 mg/kg (Female) vs. MSG + KYNA 10 mg/kg (Male)	>0.9999
		MSG + KYNA 50 mg/kg (Female) vs. MSG + KYNA 50 mg/kg (Male)	>0.9999
		MSG + KYNA 100 mg/kg (Female) vs. MSG + KYNA 100 mg/kg (Male)	0.999
		P6	
		MSG + Vehicle (Female) vs. MSG + Vehicle (Male)	>0.9999
		MSG + APV 50 mg/kg (Female) vs. MSG + APV 50 mg/kg (Male)	>0.9999
		MSG + KYNA 10 mg/kg (Female) vs. MSG + KYNA 10 mg/kg (Male)	>0.9999
		MSG + KYNA 50 mg/kg (Female) vs. MSG + KYNA 50 mg/kg (Male)	>0.9999
		MSG + KYNA 100 mg/kg (Female) vs. MSG + KYNA 100 mg/kg (Male)	>0.9999

Sex Difference – Rearing

Type of Statistical Test	Statistical Details	Multiple Comparisons	P Value
Two-way RM ANOVA with Tukey's multiple comparison test	Time factor: F(2.949, 176.9) = 1.191; Sex factor: F(11, 60) = 1.224; Time x Treatment Factor: F(55, 300) = 1.199	P1	
		MSG + Vehicle (Female) vs. MSG + Vehicle (Male)	0.998
		MSG + APV 50 mg/kg (Female) vs. MSG + APV 50 mg/kg (Male)	0.775
		MSG + KYNA 10 mg/kg (Female) vs. MSG + KYNA 10 mg/kg (Male)	0.921
		MSG + KYNA 50 mg/kg (Female) vs. MSG + KYNA 50 mg/kg (Male)	>0.9999
		MSG + KYNA 100 mg/kg (Female) vs. MSG + KYNA 100 mg/kg (Male)	>0.9999
		P2	
		MSG + Vehicle (Female) vs. MSG + Vehicle (Male)	0.997
		MSG + APV 50 mg/kg (Female) vs. MSG + APV 50 mg/kg (Male)	0.777
		MSG + KYNA 10 mg/kg (Female) vs. MSG + KYNA 10 mg/kg (Male)	>0.9999
		MSG + KYNA 50 mg/kg (Female) vs. MSG + KYNA 50 mg/kg (Male)	>0.9999
		MSG + KYNA 100 mg/kg (Female) vs. MSG + KYNA 100 mg/kg (Male)	0.913
		P3	
		MSG + Vehicle (Female) vs. MSG + Vehicle (Male)	>0.9999
		MSG + APV 50 mg/kg (Female) vs. MSG + APV 50 mg/kg (Male)	0.989
		MSG + KYNA 10 mg/kg (Female) vs. MSG + KYNA 10 mg/kg (Male)	0.864
		MSG + KYNA 50 mg/kg (Female) vs. MSG + KYNA 50 mg/kg (Male)	0.995
		MSG + KYNA 100 mg/kg (Female) vs. MSG + KYNA 100 mg/kg (Male)	0.852
		P4	
		MSG + Vehicle (Female) vs. MSG + Vehicle (Male)	0.996
		MSG + APV 50 mg/kg (Female) vs. MSG + APV 50 mg/kg (Male)	>0.9999
		MSG + KYNA 10 mg/kg (Female) vs. MSG + KYNA 10 mg/kg (Male)	0.750
		MSG + KYNA 50 mg/kg (Female) vs. MSG + KYNA 50 mg/kg (Male)	0.998
		MSG + KYNA 100 mg/kg (Female) vs. MSG + KYNA 100 mg/kg (Male)	0.956
		P5	
		MSG + Vehicle (Female) vs. MSG + Vehicle (Male)	>0.9999
		MSG + APV 50 mg/kg (Female) vs. MSG + APV 50 mg/kg (Male)	0.997
		MSG + KYNA 10 mg/kg (Female) vs. MSG + KYNA 10 mg/kg (Male)	0.999
		MSG + KYNA 50 mg/kg (Female) vs. MSG + KYNA 50 mg/kg (Male)	0.997
		MSG + KYNA 100 mg/kg (Female) vs. MSG + KYNA 100 mg/kg (Male)	0.999
		P6	
		MSG + Vehicle (Female) vs. MSG + Vehicle (Male)	>0.9999
		MSG + APV 50 mg/kg (Female) vs. MSG + APV 50 mg/kg (Male)	1.000
		MSG + KYNA 10 mg/kg (Female) vs. MSG + KYNA 10 mg/kg (Male)	>0.9999
		MSG + KYNA 50 mg/kg (Female) vs. MSG + KYNA 50 mg/kg (Male)	>0.9999
		MSG + KYNA 100 mg/kg (Female) vs. MSG + KYNA 100 mg/kg (Male)	0.891

Sex Difference – Head Scratch

Type of Statistical Test	Statistical Details	Multiple Comparisons	P Value
Two-way RM ANOVA with Tukey's multiple comparison test	Time factor: F (1.900, 114.0) = 0.1480; Sex factor: F (11, 60) = 0.9697; Time x Treatment Factor: F(55, 300) = 0.9689	P1	
		MSG + Vehicle (Female) vs. MSG + Vehicle (Male)	>0.9999
		MSG + APV 50 mg/kg (Female) vs. MSG + APV 50 mg/kg (Male)	0.995
		MSG + KYNA 10 mg/kg (Female) vs. MSG + KYNA 10 mg/kg (Male)	0.998
		MSG + KYNA 50 mg/kg (Female) vs. MSG + KYNA 50 mg/kg (Male)	0.994
		MSG + KYNA 100 mg/kg (Female) vs. MSG + KYNA 100 mg/kg (Male)	0.979
		P2	
		MSG + Vehicle (Female) vs. MSG + Vehicle (Male)	0.996
		MSG + APV 50 mg/kg (Female) vs. MSG + APV 50 mg/kg (Male)	0.980
		MSG + KYNA 10 mg/kg (Female) vs. MSG + KYNA 10 mg/kg (Male)	0.995
		MSG + KYNA 50 mg/kg (Female) vs. MSG + KYNA 50 mg/kg (Male)	0.991
		MSG + KYNA 100 mg/kg (Female) vs. MSG + KYNA 100 mg/kg (Male)	0.990
		P3	
		MSG + Vehicle (Female) vs. MSG + Vehicle (Male)	0.788
		MSG + APV 50 mg/kg (Female) vs. MSG + APV 50 mg/kg (Male)	0.995
		MSG + KYNA 10 mg/kg (Female) vs. MSG + KYNA 10 mg/kg (Male)	>0.9999
		MSG + KYNA 50 mg/kg (Female) vs. MSG + KYNA 50 mg/kg (Male)	0.984
		MSG + KYNA 100 mg/kg (Female) vs. MSG + KYNA 100 mg/kg (Male)	>0.9999
		P4	
		MSG + Vehicle (Female) vs. MSG + Vehicle (Male)	0.997
		MSG + APV 50 mg/kg (Female) vs. MSG + APV 50 mg/kg (Male)	0.998
		MSG + KYNA 10 mg/kg (Female) vs. MSG + KYNA 10 mg/kg (Male)	0.933
		MSG + KYNA 50 mg/kg (Female) vs. MSG + KYNA 50 mg/kg (Male)	1.000
		MSG + KYNA 100 mg/kg (Female) vs. MSG + KYNA 100 mg/kg (Male)	0.998
		P5	
		MSG + Vehicle (Female) vs. MSG + Vehicle (Male)	>0.9999
		MSG + APV 50 mg/kg (Female) vs. MSG + APV 50 mg/kg (Male)	>0.9999
		MSG + KYNA 10 mg/kg (Female) vs. MSG + KYNA 10 mg/kg (Male)	1.000
		MSG + KYNA 50 mg/kg (Female) vs. MSG + KYNA 50 mg/kg (Male)	0.928
		MSG + KYNA 100 mg/kg (Female) vs. MSG + KYNA 100 mg/kg (Male)	0.846
		P6	
		MSG + Vehicle (Female) vs. MSG + Vehicle (Male)	0.981
		MSG + APV 50 mg/kg (Female) vs. MSG + APV 50 mg/kg (Male)	>0.9999
		MSG + KYNA 10 mg/kg (Female) vs. MSG + KYNA 10 mg/kg (Male)	0.998
		MSG + KYNA 50 mg/kg (Female) vs. MSG + KYNA 50 mg/kg (Male)	0.994
		MSG + KYNA 100 mg/kg (Female) vs. MSG + KYNA 100 mg/kg (Male)	0.993

Sex Difference – Facial Grooming

Type of Statistical Test	Statistical Details	Multiple Comparisons	P Value
Two-way RM ANOVA with Tukey's multiple comparison test	Time factor: F(3.317, 199.0) = 1.417; Sex factor: F(11, 60) = 1.001; Time x Treatment Factor: F(55, 300) = 0.9307	P1	
		MSG + Vehicle (Female) vs. MSG + Vehicle (Male)	>0.9999
		MSG + APV 50 mg/kg (Female) vs. MSG + APV 50 mg/kg (Male)	0.955
		MSG + KYNA 10 mg/kg (Female) vs. MSG + KYNA 10 mg/kg (Male)	0.996
		MSG + KYNA 50 mg/kg (Female) vs. MSG + KYNA 50 mg/kg (Male)	>0.9999
		MSG + KYNA 100 mg/kg (Female) vs. MSG + KYNA 100 mg/kg (Male)	0.986
		P2	
		MSG + Vehicle (Female) vs. MSG + Vehicle (Male)	>0.9999
		MSG + APV 50 mg/kg (Female) vs. MSG + APV 50 mg/kg (Male)	>0.9999
		MSG + KYNA 10 mg/kg (Female) vs. MSG + KYNA 10 mg/kg (Male)	>0.9999
		MSG + KYNA 50 mg/kg (Female) vs. MSG + KYNA 50 mg/kg (Male)	>0.9999
		MSG + KYNA 100 mg/kg (Female) vs. MSG + KYNA 100 mg/kg (Male)	0.990
		P3	
		MSG + Vehicle (Female) vs. MSG + Vehicle (Male)	0.618
		MSG + APV 50 mg/kg (Female) vs. MSG + APV 50 mg/kg (Male)	>0.9999
		MSG + KYNA 10 mg/kg (Female) vs. MSG + KYNA 10 mg/kg (Male)	>0.9999
		MSG + KYNA 50 mg/kg (Female) vs. MSG + KYNA 50 mg/kg (Male)	>0.9999
		MSG + KYNA 100 mg/kg (Female) vs. MSG + KYNA 100 mg/kg (Male)	>0.9999
		P4	
		MSG + Vehicle (Female) vs. MSG + Vehicle (Male)	>0.9999
		MSG + APV 50 mg/kg (Female) vs. MSG + APV 50 mg/kg (Male)	>0.9999
		MSG + KYNA 10 mg/kg (Female) vs. MSG + KYNA 10 mg/kg (Male)	0.768
		MSG + KYNA 50 mg/kg (Female) vs. MSG + KYNA 50 mg/kg (Male)	>0.9999
		MSG + KYNA 100 mg/kg (Female) vs. MSG + KYNA 100 mg/kg (Male)	0.996
		P5	
		MSG + Vehicle (Female) vs. MSG + Vehicle (Male)	>0.9999
		MSG + APV 50 mg/kg (Female) vs. MSG + APV 50 mg/kg (Male)	>0.9999
		MSG + KYNA 10 mg/kg (Female) vs. MSG + KYNA 10 mg/kg (Male)	>0.9999
		MSG + KYNA 50 mg/kg (Female) vs. MSG + KYNA 50 mg/kg (Male)	0.999
		MSG + KYNA 100 mg/kg (Female) vs. MSG + KYNA 100 mg/kg (Male)	0.869
		P6	
		MSG + Vehicle (Female) vs. MSG + Vehicle (Male)	0.996
		MSG + APV 50 mg/kg (Female) vs. MSG + APV 50 mg/kg (Male)	0.996
		MSG + KYNA 10 mg/kg (Female) vs. MSG + KYNA 10 mg/kg (Male)	0.900
		MSG + KYNA 50 mg/kg (Female) vs. MSG + KYNA 50 mg/kg (Male)	0.872
		MSG + KYNA 100 mg/kg (Female) vs. MSG + KYNA 100 mg/kg (Male)	>0.9999

Sex Difference – Mechanical Threshold

Type of Statistical Test	Statistical Details	Multiple Comparisons	P Value
Two-way RM ANOVA with Tukey's multiple comparison test	Time factor: F(3.177, 190.6) = 0.6540; Sex factor: F(11, 60) = 0.4609; Time x Treatment Factor: F(55, 300) = 0.8679	P1	
		MSG + Vehicle (Female) vs. MSG + Vehicle (Male)	0.921
		MSG + APV 50 mg/kg (Female) vs. MSG + APV 50 mg/kg (Male)	0.656
		MSG + KYNA 10 mg/kg (Female) vs. MSG + KYNA 10 mg/kg (Male)	>0.9999
		MSG + KYNA 50 mg/kg (Female) vs. MSG + KYNA 50 mg/kg (Male)	1.000
		MSG + KYNA 100 mg/kg (Female) vs. MSG + KYNA 100 mg/kg (Male)	0.549
		P2	
		MSG + Vehicle (Female) vs. MSG + Vehicle (Male)	0.935
		MSG + APV 50 mg/kg (Female) vs. MSG + APV 50 mg/kg (Male)	>0.9999
		MSG + KYNA 10 mg/kg (Female) vs. MSG + KYNA 10 mg/kg (Male)	0.999
		MSG + KYNA 50 mg/kg (Female) vs. MSG + KYNA 50 mg/kg (Male)	0.999
		MSG + KYNA 100 mg/kg (Female) vs. MSG + KYNA 100 mg/kg (Male)	0.834
		P3	
		MSG + Vehicle (Female) vs. MSG + Vehicle (Male)	>0.9999
		MSG + APV 50 mg/kg (Female) vs. MSG + APV 50 mg/kg (Male)	>0.9999
		MSG + KYNA 10 mg/kg (Female) vs. MSG + KYNA 10 mg/kg (Male)	>0.9999
		MSG + KYNA 50 mg/kg (Female) vs. MSG + KYNA 50 mg/kg (Male)	>0.9999
		MSG + KYNA 100 mg/kg (Female) vs. MSG + KYNA 100 mg/kg (Male)	0.707
		P4	
		MSG + Vehicle (Female) vs. MSG + Vehicle (Male)	>0.9999
		MSG + APV 50 mg/kg (Female) vs. MSG + APV 50 mg/kg (Male)	>0.9999
		MSG + KYNA 10 mg/kg (Female) vs. MSG + KYNA 10 mg/kg (Male)	>0.9999
		MSG + KYNA 50 mg/kg (Female) vs. MSG + KYNA 50 mg/kg (Male)	0.996
		MSG + KYNA 100 mg/kg (Female) vs. MSG + KYNA 100 mg/kg (Male)	0.771
		P5	
		MSG + Vehicle (Female) vs. MSG + Vehicle (Male)	0.930
		MSG + APV 50 mg/kg (Female) vs. MSG + APV 50 mg/kg (Male)	0.906
		MSG + KYNA 10 mg/kg (Female) vs. MSG + KYNA 10 mg/kg (Male)	>0.9999
		MSG + KYNA 50 mg/kg (Female) vs. MSG + KYNA 50 mg/kg (Male)	>0.9999
		MSG + KYNA 100 mg/kg (Female) vs. MSG + KYNA 100 mg/kg (Male)	0.989
		P6	
		MSG + Vehicle (Female) vs. MSG + Vehicle (Male)	0.935
		MSG + APV 50 mg/kg (Female) vs. MSG + APV 50 mg/kg (Male)	>0.9999
		MSG + KYNA 10 mg/kg (Female) vs. MSG + KYNA 10 mg/kg (Male)	>0.9999
		MSG + KYNA 50 mg/kg (Female) vs. MSG + KYNA 50 mg/kg (Male)	0.867
		MSG + KYNA 100 mg/kg (Female) vs. MSG + KYNA 100 mg/kg (Male)	>0.9999

Sex Difference – Lying-on-belly

Type of Statistical Test	Statistical Details	Multiple Comparisons	P Value
Two-way RM ANOVA with Tukey's multiple comparison test	Time factor: F(3.169, 190.1) = 19.23; Sex factor: F(11, 60) = 3.218; Time x Treatment Factor: F(55, 300) = 2.282	P1	
		MSG + Vehicle (Male) vs. MSG + Vehicle (Female)	0.8373
		MSG + APV 50 mg/kg (Male) vs. MSG + APV 50 mg/kg (Female)	>0.9999
		MSG + KYNA 10 mg/kg (Male) vs. MSG + KYNA 10 mg/kg (Female)	0.999
		MSG + KYNA 50 mg/kg (Male) vs. MSG + KYNA 50 mg/kg (Female)	>0.9999
		MSG + KYNA 100 mg/kg (Male) vs. MSG + KYNA 100 mg/kg (Female)	0.0352
		P2	
		MSG + Vehicle (Male) vs. MSG + Vehicle (Female)	0.9988
		MSG + APV 50 mg/kg (Male) vs. MSG + APV 50 mg/kg (Female)	>0.9999
		MSG + KYNA 10 mg/kg (Male) vs. MSG + KYNA 10 mg/kg (Female)	0.3369
		MSG + KYNA 50 mg/kg (Male) vs. MSG + KYNA 50 mg/kg (Female)	0.9806
		MSG + KYNA 100 mg/kg (Male) vs. MSG + KYNA 100 mg/kg (Female)	0.7537
		P3	
		MSG + Vehicle (Male) vs. MSG + Vehicle (Female)	>0.9999
		MSG + APV 50 mg/kg (Male) vs. MSG + APV 50 mg/kg (Female)	>0.9999
		MSG + KYNA 10 mg/kg (Male) vs. MSG + KYNA 10 mg/kg (Female)	0.9758
		MSG + KYNA 50 mg/kg (Male) vs. MSG + KYNA 50 mg/kg (Female)	>0.9999
		MSG + KYNA 100 mg/kg (Male) vs. MSG + KYNA 100 mg/kg (Female)	>0.9999
		P4	
		MSG + Vehicle (Male) vs. MSG + Vehicle (Female)	>0.9999
		MSG + APV 50 mg/kg (Male) vs. MSG + APV 50 mg/kg (Female)	>0.9999
		MSG + KYNA 10 mg/kg (Male) vs. MSG + KYNA 10 mg/kg (Female)	>0.9999
		MSG + KYNA 50 mg/kg (Male) vs. MSG + KYNA 50 mg/kg (Female)	0.9882
		MSG + KYNA 100 mg/kg (Male) vs. MSG + KYNA 100 mg/kg (Female)	>0.9999
		P5	
		MSG + Vehicle (Male) vs. MSG + Vehicle (Female)	>0.9999
		MSG + APV 50 mg/kg (Male) vs. MSG + APV 50 mg/kg (Female)	>0.9999
		MSG + KYNA 10 mg/kg (Male) vs. MSG + KYNA 10 mg/kg (Female)	>0.9999
		MSG + KYNA 50 mg/kg (Male) vs. MSG + KYNA 50 mg/kg (Female)	>0.9999
		MSG + KYNA 100 mg/kg (Male) vs. MSG + KYNA 100 mg/kg (Female)	>0.9999
		P6	
		MSG + Vehicle (Male) vs. MSG + Vehicle (Female)	>0.9999
		MSG + APV 50 mg/kg (Male) vs. MSG + APV 50 mg/kg (Female)	0.9985
		MSG + KYNA 10 mg/kg (Male) vs. MSG + KYNA 10 mg/kg (Female)	>0.9999
		MSG + KYNA 50 mg/kg (Male) vs. MSG + KYNA 50 mg/kg (Female)	>0.9999
		MSG + KYNA 100 mg/kg (Male) vs. MSG + KYNA 100 mg/kg (Female)	>0.9999
